# ZBP1 Drives CD8^+^ T cell-mediated anti-tumor immunity in head and neck squamous cell carcinoma

**DOI:** 10.1371/journal.pgen.1012107

**Published:** 2026-05-26

**Authors:** Yu Min, Ge Song, Lianlian Yang, Ling He, Shihong Xu, Kun Gao, Zheran Liu, Xingchen Peng, Lei Dai

**Affiliations:** 1 Department of Biotherapy, Cancer Center, West China Hospital, Sichuan University, ‌‌Chengdu, China; 2 Department of Oncology, The General Hospital of Western Theater Command, Chengdu, China; 3 Department of Biotherapy, Cancer Center and State Key Laboratory of Biotherapy,‌‌ West China Hospital, Sichuan University, Chengdu, Sichuan, China; Cornell University College of Veterinary Medicine, UNITED STATES OF AMERICA

## Abstract

Head and neck squamous cell carcinoma (HNSCC) frequently resists PD-1 blockade due to an immunologically “cold” tumor microenvironment (TME). Here, we identify Z-DNA binding protein 1 (ZBP1) as a key immunoregulator that reprograms immune-suppressive TMEs. Integrated TCGA/SangerBox analyses revealed ZBP1 as a hub gene strongly correlated with cytotoxic CD8^+^ T cells (r = 0.48, *p* < 0.0001) and M1 macrophages (r = 0.39, *p* < 0.0001). Multi-model validation in 92 HNSCC specimens revealed elevated ZBP1 expression versus normal tissues (*p* < 0.01), co-localized with infiltrating CD8^+^/CD4^+^ T cells and CD68^+^ macrophages through multiplex immunofluorescence. Clinically, high ZBP1 predicted improved survival (HR = 0.61 for overall survival; HR = 0.45 for disease specific survival; *p* < 0.0001) and early-stage presentation (*p* = 0.004). Mechanistically, ZBP1 overexpression in SCC-7/MOC2 models suppressed tumor growth while enhancing IFN-γ^+^ CD8^+^ T cell activation and reducing M2 polarization (CD206^+^: 16.91% vs 38.19% in ZBP1-high vs control, *p* < 0.001). Single-cell transcriptomics uncovered ZBP1-driven TME remodeling through chemokine signaling networks and expanded effector T cell compartments, validated by 1.49-fold increased CD8^+^ T cell infiltration via flow cytometry. Spatial analysis revealed ZBP1 overexpression amplified immune cell crosstalk (1.65-fold interaction increase, *p* < 0.001), upregulating CD8^+^ T cell chemotaxis (CXCR3/CCR5-CCL5 axis) and effector functions (*p* < 0.0001). Concurrently, it suppressed immunosuppressive pathways through metabolic reprogramming, establishing ZBP1 as a dual regulator synchronizing lymphocyte recruitment and myeloid suppression. Our integrative approach bridges computational biology with functional validation, demonstrating ZBP1’s capacity to convert “cold” tumors into immunologically active niches. This work positions ZBP1 as both a stratification biomarker for checkpoint inhibitor response and a therapeutic target for TME reprogramming in HNSCC.

## Author summary

Head and neck squamous cell carcinoma is a common and aggressive cancer. A major challenge in its treatment is that many tumors are “cold,” meaning they are not easily recognized or attacked by the body’s immune system, leading to poor responses to modern immunotherapies. In this study, we identified a protein called ZBP1 that acts as a master switch to transform these cold tumors into “hot,” immune-active environments. We found that patients whose tumors have high levels of ZBP1 have more cancer-fighting immune cells inside the tumor, experience earlier-stage disease, and live longer. Using mouse models and advanced molecular profiling, we discovered that ZBP1 works by two key mechanisms: first, it recruits and activates anti-tumor immune cells, particularly CD8^+^ “killer” T cells; and second, it suppresses immune-suppressive cell types and pathways. Our findings bridge computational analysis with experimental validation, suggesting that ZBP1 could serve both as a biomarker to predict which patients may respond to immunotherapy and as a promising therapeutic target to reprogram the tumor microenvironment and improve outcomes in head and neck cancer.

## 1. Background

Head and neck squamous cell carcinoma (HNSCC), a frequently occurring epithelial malignancy originating in the mucosal surfaces of the oral cavity, oropharynx, hypopharynx, and laryngeal regions, remains a major worldwide health challenge [1]. Global Cancer Observatory data indicate approximately 890,000 incident cases and 450,000 cancer-related fatalities annually, positioning HNSCC as the seventh most prevalent malignancy worldwide [2]. Established risk factors comprise tobacco use, alcohol consumption, betel quid chewing in endemic areas, and human papillomavirus (HPV) infection, the latter increasingly implicated in oropharyngeal cancer among younger populations in North America and Northern Europe [3,4]. While early-stage HNSCC patients achieve favorable outcomes with surgery or radiotherapy (70–90% cure rates), over 60% of cases are diagnosed at advanced stages (III/IV), characterized by extensive local invasion, lymph node metastasis, and dismal 5-year survival rates (<50%) [5,6]. Systemic therapies for recurrent/metastatic HNSCC remain limited, with platinum, taxanes, 5-fluorouracil, and cetuximab offering modest efficacy. Therapeutic strategies involving programmed death-1/programmed death-ligand 1 (PD-1/PD-L1)-directed immune checkpoint inhibitors (ICIs) have fundamentally altered clinical management algorithms, as demonstrated in landmark trials (KEYNOTE-012, CheckMate 141, KEYNOTE-048), yet only 20–30% of patients derive durable clinical benefits [7–12].

The tumor immune microenvironment (TIME) critically determines ICI responsiveness. HNSCC exhibits heterogeneous immune infiltration patterns, classified as inflamed, immune-excluded, or immune-desert phenotypes [13,14]. Although HPV-positive HNSCC demonstrates superior responses to various therapies, including immunotherapy, and improved prognosis compared to HPV-negative counterparts [15–19], evidence indicates that HPV-negative HNSCC patients achieve comparable clinical outcomes when matched for tumor-infiltrating CD8^+^ T cells proportions [20–22].CD8^+^ T cells mediate antitumor immunity via direct cytotoxicity and cytokine secretion [23], while tumor-associated macrophages (TAMs), skewed toward immunosuppressive M2 phenotypes in HNSCC, secrete IL-10 and TGF-β to dampen immune activation [24–26]. Strategies to enhance CD8^+^ T cells infiltration and reprogram TAMs are thus urgently needed to broaden ICI efficacy.

Z-DNA binding protein 1 (ZBP1), functioning dually as an intracellular pattern recognition receptor and interferon-inducible effector, has emerged as a pivotal regulator of antitumor immunity [27,28]. ZBP1 activation triggers necroptosis via RIPK3-MLKL signaling and induces type I interferon production through TBK1-IRF3 pathways, fostering dendritic cell (DC) maturation and CD8^+^ T cells priming [29–33]. Preclinical studies reveal context-dependent roles: ZBP1 suppresses colorectal and melanoma growth but promotes myeloma progression [34–36]. Notably, radiation-induced ZBP1 activation enhances systemic antitumor immunity by recruiting CD8^+^ T cells and DCs, while curaxin CBL0137 reactivates immune-excluded tumors by stimulating ZBP1-dependent interferon responses in stromal cells [29,37]. Despite these advances, ZBP1’s function in HNSCC remains unexplored.

Here, we identify ZBP1 as a novel immune-associated biomarker exhibiting a significant positive association with CD8^+^ T cell tumor infiltration and improved clinical prognosis in HNSCC. By integrating multi-omics profiling, immunohistochemical mapping, and functional validation, we elucidate ZBP1’s spatial organization and its regulatory role in modulating tumor proliferation, apoptotic resistance, and immune microenvironment remodeling. Strikingly, employing ZBP1-overexpressing murine tumor models coupled with single-cell transcriptomic analysis, we mechanistically uncover ZBP1-driven reprogramming of the TIME, characterized by augmented cytotoxic lymphocyte recruitment and suppression of pro-tumorigenic M2-like tumor-associated macrophage polarization. These findings establish ZBP1 not only as a prognostic indicator but also as a druggable target for alleviating immunosuppressive barriers in HNSCC, providing a molecular rationale for synergistic therapeutic approaches with ICIs.

## 2. Results

### 2.1. Bioinformatic Identification of ZBP1 as a Multifaceted Immune Infiltration Regulator in HNSCC

**Fig 1:**
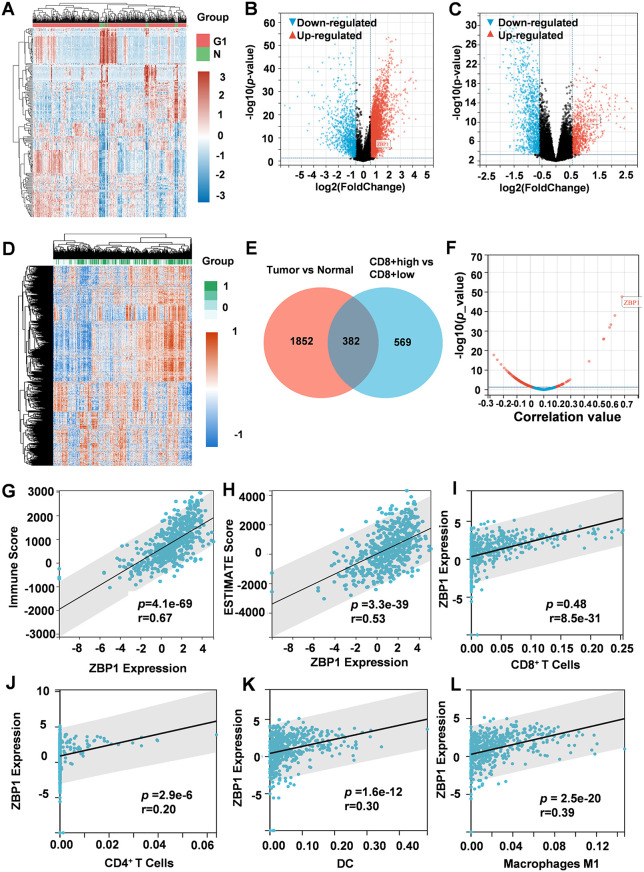
Multi-dimensional characterization of ZBP1 as an immune infiltration-associated regulator in HNSCC. **(A)** Hierarchical clustering of heatmap distinguishing HNSCC (G1) from normal mucosa (N) in TCGA cohort. **(B)** Volcano plot identifying tumor-specific transcriptional alterations between HNSCC and matched peritumoral tissues. **(C)** CD8^+^ T cell infiltration-dependent gene expression signatures revealed by comparative analysis of high vs low lymphocyte-infiltrated HNSCC specimens. **(D**) Heatmap of DEGs stratified by CD8^+^ T cell infiltration status. **(E)** Venn diagram showing 382 overlapping DEGs between tumor-normal and immune infiltration contrasts. **(F)** Correlation between overlapping genes and activated CD8^+^ T cell signatures (Spearman’s test). **(G-L)** ZBP1 expression correlates with TIME features: **(G)** Immune Score (r = 0.67, *p* < 0.001) **(H)** ESTIMATE Score (r = 0.53, *p* < 0.001) **(I)** CD8^+^ T cells (*r* = 0.48, *p* < 0.001) **(J)** CD4^+^ T cells (*r* = 0.20, *p* < 0.001) **(K)** Dendritic cells (*r* = 0.30, *p* < 0.001) **(L)** M1 macrophages (*r* = 0.39, *p* < 0.001).

Integrated analysis of TCGA-HNSCC datasets revealed ZBP1 as a central hub gene associated with cytotoxic and innate immune cell infiltration. Comparative transcriptomics identified 2,234 differentially expressed genes (DEGs) between tumor and adjacent normal tissues (Fig 1A-1B), with 951 DEGs further stratified by CD8+ T cells infiltration levels (Fig 1C-1D). Intersection analysis yielded 382 overlapping genes (Fig 1E-1F).

Expanding this finding, ZBP1 expression positively correlated with global immune infiltration metrics, including Immune score (*r* = 0.67; Fig 1G) and ESTIMATE stromal-immune scores (*r* = 0.53; Fig 1H). At the cellular level, ZBP1 showed significant associations with CD8^+^ T cells (*r* = 0.48), CD4^+^ T cells (*r* = 0.20), dendritic cells (*r* = 0.30), and M1-polarized tumor-associated macrophages (*r* = 0.39), all *p* < 0.0001 (Fig 1I-L). Collectively, these findings establish ZBP1 as a pan-immunologic coordinator bridging nucleic acid sensing with adaptive (CD8^+^/CD4^+^ T cells) and innate (DC/M1 macrophages) anti-tumor immunity in HNSCC.

### 2.2. ZBP1 Overexpression in HNSCC Associates with Immune Cell Infiltration

**Fig 2:**
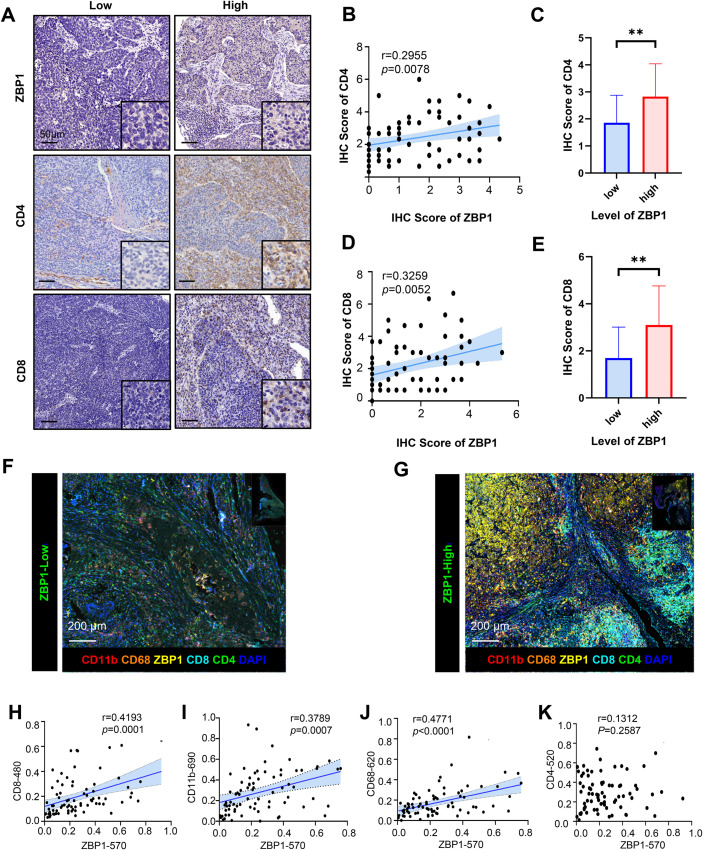
ZBP1 Expression in HNSCC Tissues and Its Correlation with Immune Cell Infiltration. **(A)** Representative immunohistochemical co-staining of ZBP1 with CD8^+^ or CD4^+^ T cells in HNSCC tissue microarray (TMA). **(B)** Positive correlation between ZBP1 expression and CD8^+^ T cell infiltration (Pearson’s r = 0.30, p = 0.0078). Student’s-t test, **p < 0.01.Data: mean ± SEM. **(C)** CD4^+^ T cell infiltration scores stratified by ZBP1 expression subgroups (ZBP1-high: score ≥3; ZBP1-low: score ≤2). Student’s -t test, **p < 0.01.**(D)** Correlation of ZBP1 expression with CD8^+^ T cell infiltration (Pearson’s r = 0.30, p = 0.0078). **(E)** CD8^+^ T cell infiltration scores across ZBP1 expression subgroups.Student’s-t test, **p < 0.01.Data: mean ± SEM. **(F)** Multiplex immunofluorescence images of HNSCC tissues. Markers: ZBP1 (yellow), CD8 (cyan), CD4 (green), CD11b (red), CD68 (orange), nuclei (DAPI, blue) in ZBP1 low group. **(G)** Multiplex immunofluorescence images of HNSCC tissues. Markers: ZBP1 (yellow), CD8 (cyan), CD4 (green), CD11b (red), CD68 (orange), nuclei (DAPI, blue) in ZBP1 high group. **(H-J)** Correlations between ZBP1^+^ cell density and infiltration levels of CD8^+^ T cells (*r* = 0.42), CD11b^+^ cells (*r* = 0.38), and CD68^+^ macrophages (*r* = 0.48). **(K)** No significant correlation between ZBP1^+^ cells and CD4^+^ T cells. Fluorescence counts normalized to DAPI^+^ nuclei per TMA core. Scale bars: 50 μm **(A)**, 200μm (**F**, **G**).

Building upon bioinformatic predictions, we systematically validated ZBP1’s immunomodulatory roles across multi-omics and clinical cohorts. Pan-cancer analysis revealed elevated ZBP1 expression in HNSCC, lung adenocarcinoma (LUAD), breast invasive carcinoma (BRCA), and mixed-type renal carcinoma (KIPAN) compared to adjacent normal tissues (S1A Fig). IHC staining of 92 HNSCC specimens confirmed tumor-specific ZBP1 upregulation versus paired normal epithelia (S1B and S1C Fig). Critically, ZBP1 expression showed concordance with cytotoxicity and helper T cell infiltration. IHC co-staining revealed that ZBP1 intensity was positively correlated with CD8^+^ T cells density (r = 0.326) and CD4^+^ T cell abundance (r = 0.296; Fig 2A, 2B and 2D). Stratified analyses further revealed a high rate of CD8^+^ T cells and CD4^+^ T cell infiltration in ZBP1 high-level tumors (score ≥3) compared with ZBP1 low-level tumors (score ≤2; *p* < 0.05 for both; Fig 2C and 2E). Multiplex immunofluorescence revealed that ZBP1-high regions spatially colocalized with dense infiltration of CD8^+^ T cells, CD11b^+^ myeloid cells, and CD68^+^ macrophages within the TIME (Fig 2F-2G). Quantitative spatial analysis confirmed strong correlations between ZBP1^+^ cell density and CD8^+^ T cells (r = 0.419), CD11b^+^ myeloid cells (*r* = 0.379), and CD68^+^ macrophages (*r* = 0.477), but not CD4^+^ T cells (Fig 2H-2K). These results further confirmed the positive correlation between ZBP1 expression and CD8^+^ T cells infiltration.

### 2.3. Functional Enrichment and Prognostic Significance of ZBP1 in HNSCC

**Fig 3:**
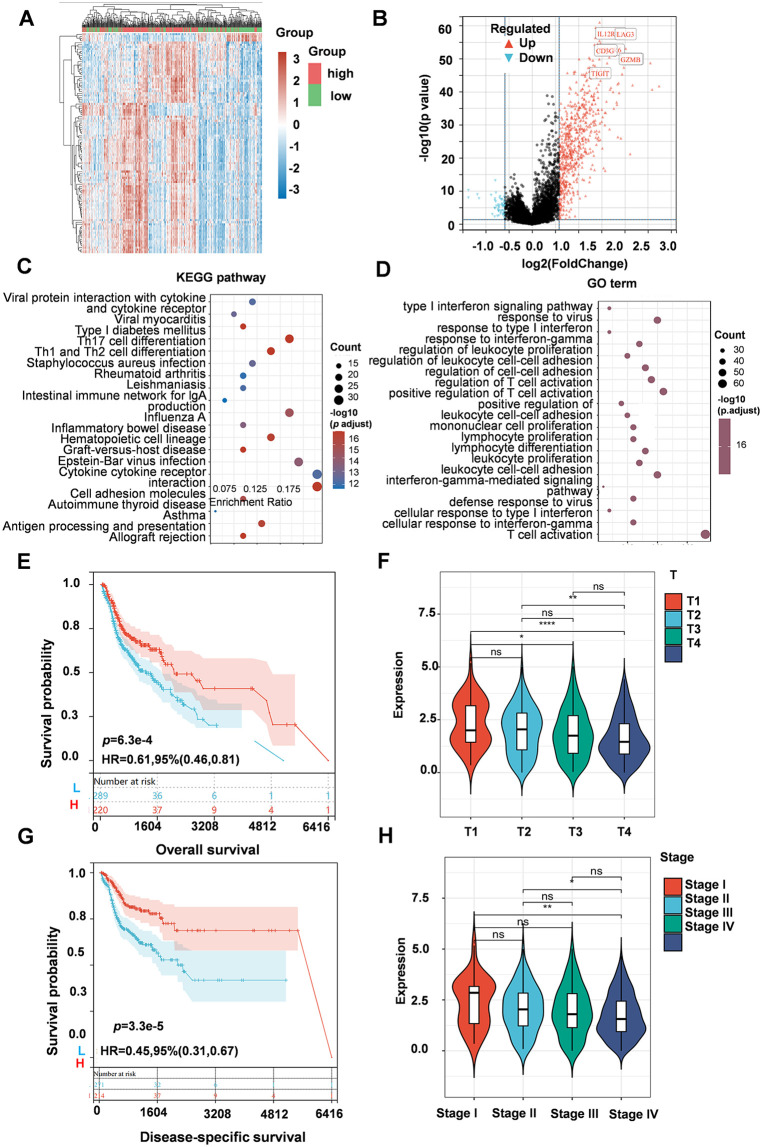
Analysis of ZBP1-associated signaling pathways, biological processes, and prognostic value in HNSCC. **(A)** Heatmap of differentially expressed genes (DEGs) between ZBP1-high (high) and ZBP1-low (low) tissues from the TCGA-HNSCC cohort. **(B)** Differential gene expression analysis (volcano plot) between ZBP1-high and ZBP1-low HNSCC cohorts. **(C)** KEGG pathway enrichment of upregulated genes in ZBP1-high tumors. Bubble size and color intensity correspond to gene counts and −log_10_(*p*-value), respectively. **(D)** GO biological process analysis of upregulated genes in ZBP1-high tumors, with bubble metrics as in panel **C. (E)** Overall survival (OS) comparison between ZBP1-high (H) and ZBP1-low (L) cohorts (n = 509). **(F)** ZBP1 expression stratified by T-stage classification. **(G)** Disease-specific survival (DSS) analysis across ZBP1 expression groups (n = 485). **(H)** ZBP1 expression variations among pathological stages. Data expressed as mean ± SEM. Statistical methods: Log-rank test **(E, G)**; One-way ANOVA test **(F, H)**. ZBP1 expression thresholds: high = top 50%; low = bottom 50%. Significance levels: *p < 0.05, **p < 0.01, ****p < 0.0001.

Bioinformatic interrogation of TCGA-HNSCC cohorts revealed ZBP1 as a dual-functional regulator bridging immunologic pathways and clinical outcomes. Differential expression analysis between ZBP1-high (top quartile) and -low (bottom quartile) tumors identified 249 DEGs (245 upregulated, 4 downregulated; Fig 3A**-**3B). KEGG enrichment of upregulated genes highlighted viral response pathways (influenza A; EBV infection) and immune coordination processes (T cell differentiation; cytokine-cytokine receptor interaction; **Fig 3C**). GO analysis further linked ZBP1 to T cell activation regulation, antiviral defense, and type I interferon signaling (**Fig 3D**). Clinically, elevated ZBP1 expression correlated with improved survival, conferring 39% reduced mortality risk (Overall Survival: HR = 0.61, 95% CI 0.46–0.81; *p* < 0.0001; **Fig 3E**) and 55% lower disease-specific survival (DSS: HR = 0.45, 95% CI 0.31–0.67; *p* < 0.0001; **Fig 3G**). Strikingly, ZBP1 levels exhibited early clinical stage, with highest expression in Stage I and T1 tumors versus minimal detection in Stage IV and T4 lesions (**Fig 3F and 3H**). These data establish ZBP1 as both a mechanistic orchestrator of antitumor immunity and an independent prognostic biomarker in HNSCC, whose progressive loss during tumor evolution may drive immune escape.

### 2.4. Spatial and cubcellular ZBP1 Architecture in HNSCC

**Fig 4:**
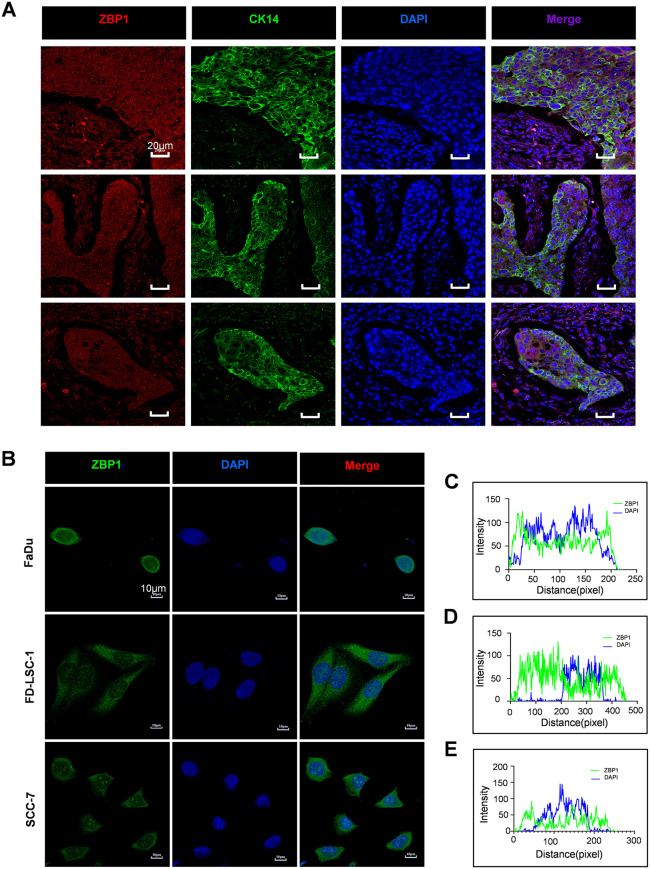
Multiscale ZBP1 Localization in HNSCC. **(A)** Dual tumor-stromal ZBP1 expression in clinical HNSCC. Immunofluorescence co-staining of human HNSCC tissues showing ZBP1 (red) co-expression with epithelial marker CK14 (green) in tumor nests and stromal compartments. Insets: High-magnification views of ZBP1^+^ tumor cells (CK14^+^ZBP1^+^) and stromal immune infiltrates (CK14-ZBP1^+^). Nuclei counterstained with DAPI (blue). **(B-E)** Evolutionary divergence in ZBP1 subcellular partitioning. **(B)** Representative images of ZBP1 (green) localization in human (FaDu, FD-LSC-1) and murine (SCC-7) HNSCC cell lines. **(C-E)** Quantitative individual-cell fluorescence intensity analysis of cytoplasmic vs nuclear ZBP1. Scale bars: 20 μm **(A)**, 10 μm **(B)**.

Building upon the pan-immunologic regulatory role of ZBP1 identified in bioinformatics and IHC analyses, we performed dual immunofluorescence co-staining to resolve its cellular distribution in clinical HNSCC specimens. ZBP1 was ubiquitously expressed across tumor epithelial compartments (Pan-CK^+^) and stromal niches, including vimentin^+^ cancer-associated fibroblasts and CD45^+^ leukocyte infiltrates. Notably, colocalization analysis revealed that many ZBP1^+^ tumor cells (CK14^+^) exhibited concurrent nuclear/cytoplasmic ZBP1 signals, while stromal ZBP1 was predominantly localized to immune infiltrates (Fig 4A). Immunofluorescence quantification across three HNSCC cell lines revealed cell type-specific compartmentalization of ZBP1. In FaDu cells, ZBP1 exhibited balanced cytoplasmic-nuclear distribution (Fig 4B and 4C). In contrast, FD-LSC-1 cells showed predominant cytoplasmic retention, with minimal nuclear signal (Fig 4B and 4D). Murine SCC-7 cells demonstrated exclusive cytoplasmic accumulation, a pattern conserved across multiple passages (Fig 4B and 4E). These findings suggest progressive cytoplasmic sequestration of ZBP1 in advanced HNSCC models, potentially linked to its functional divergence between tumor-intrinsic regulation and immune crosstalk identified in clinical specimens.

### 2.5. Generation and functional characterization of ZBP1-Knockout HNSCC cells

**Fig 5:**
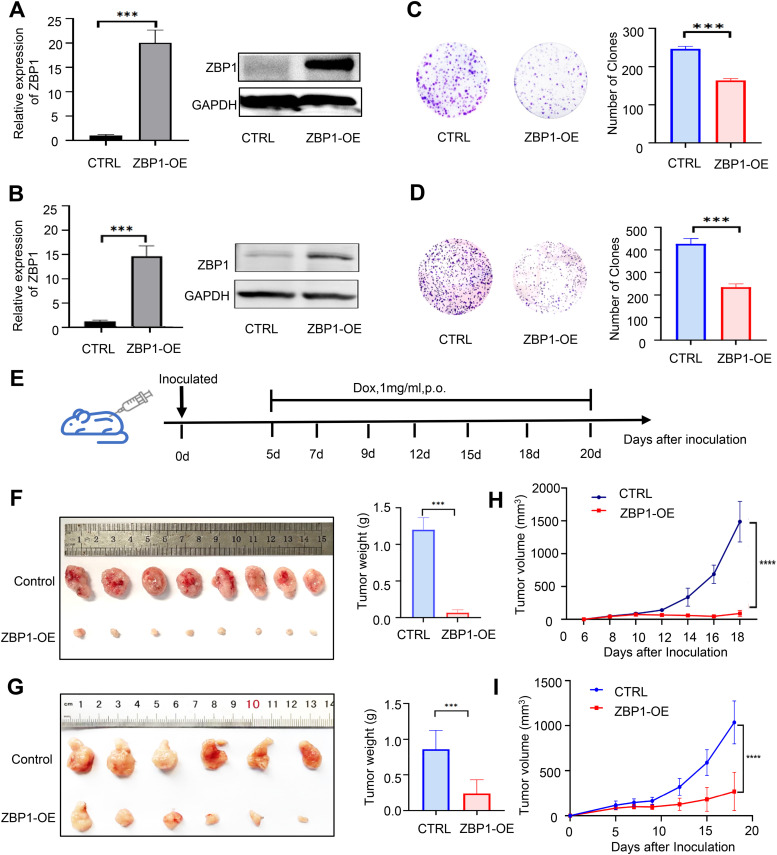
Functional consequences of ZBP1 overexpression in HNSCC models. **(A-B)**. ZBP1 mRNA (qPCR) and Western blot (WB) levels in **(A)** SCC-7 and **(B)** MOC2 cells: *** *p* < 0.001 (n = 3) **(C-D)**. Colony formation assays of **(C)** SCC-7 and **(D)** MOC2 cells (14 days): Right: Quantified colony numbers (***p < 0.001, n = 6); Left: Representative crystal violet staining. **(E)**Schematic diagram of inoculation, administration, and measurement of SCC-7/MOC2 cells in mice. (F-G) Compare the volume of subcutaneous transplanted tumors between two groups of mice, and compare the ‌‌volume and tumor mass after isolating the subcutaneous tumors. **(F)**MOC2 (n = 8); **(G)** SCC-7 (n = 6). **(H, I)** Tumor growth curves . (Statistical notation: Student-t test, *** *p* < 0.001, ****p < 0.0001. Group labels: CTRL (empty vector), ZBP1-OE (ZBP1-overexpressing cells).

To investigate the functional role of ZBP1 in HNSCC, stable ZBP1-overexpressing cell lines (ZBP1-OE) were generated in SCC-7 and MOC2 cells, with qPCR and Western blot confirming elevated ZBP1 expression compared to vector controls (Figs 5A, 5B, and S2-S3). Functional assays demonstrated diminished colony-forming capacity in ZBP1-OE clones relative to parental cells (Fig 5C and 5D). Flow cytometry revealed increased apoptotic cell proportions (Annexin V^+^/PI^+^ and Annexin V^+^/PI^−^ populations) in ZBP1-OE groups (S4 Fig). Furthermore, CRISPR/Cas9-mediated ZBP1 knockout (ZBP1-KO) SCC-7 cell lines were established using sgRNA targeting exon regions of the ZBP1 gene (S5A, S6). Monoclonal populations were screened and validated through T7 endonuclease assays, Sanger sequencing, qPCR, and Western blotting. Genomic editing was confirmed by T7EI cleavage patterns and sequence chromatograms (S5B and S5C Fig). Radiation-induced ZBP1 expression was markedly reduced at both transcriptional and protein levels in ZBP1-KO clones (KO1, KO2) compared to wild-type controls ((S5D and S5E Fig). Functional assessment of ZBP1-KO cells revealed no alterations in proliferation rates (CCK-8 assay), colony-forming capacity, or apoptosis levels (Annexin V/PI staining) relative to parental SCC-7 cells (S7 and S8 Figs). Given the tumor-suppressive effects observed in vitro, we next evaluated ZBP1’s impact on *in vivo* tumor growth (Fig 5E). Subcutaneous xenografts generated with Dox-inducible ZBP1-overexpressing SCC-7 cells and Moc2 cells exhibited reduced tumor volumes compared to control groups, with sustained suppression observed until endpoint (Fig 5F and 5G). Terminal tumor volume confirmed diminished growth in ZBP1-OE cohorts (Fig 5H and 5I). These results indicated that ZBP1 impairs tumor growth *in vitro* and *in vivo*.

### 2.6. ZBP1 reprograms tumor immune microenvironment via CD8^+^ T cells activation

**Fig 6:**
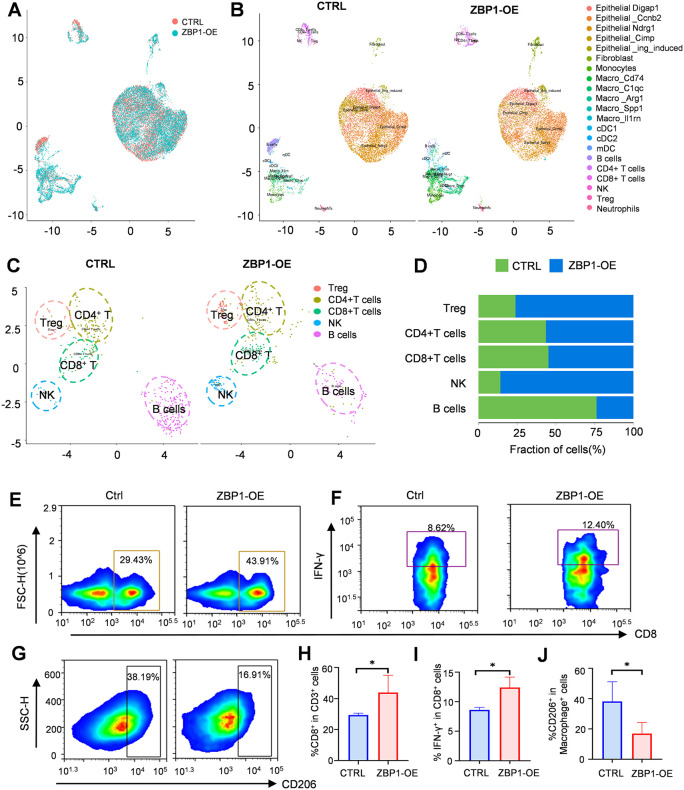
ZBP1 overexpression remodels immune cell infiltration in SCC-7 tumors. **(A)** UMAP visualization of scRNA-seq data color-coded by experimental groups (Ctrl vs ZBP1-OE). **(B)** Single-cell RNA sequencing (scRNA-seq) clustering analysis displaying canonical cell type markers across clusters. **(C)** Feature plots identify five major immune cell clusters in ZBP1-OE and CTRL subcutaneous tumors. **(D**) Differential immune cell infiltration analysis quantified by cell type proportions. **(E-G)** Flow cytometry of SCC-7 tumor-infiltrating immune cells: (E/H): CD8^+^ T cell percentages (Ctrl vs OE), (F/I): CD8^+^IFN-γ^+^T cell proportions (G/J), M2 (CD11b^+^F4/80^+^CD206^+^) macrophage proportions (Statistical notation: Student-t test, *p < 0.05).

To determine the underlying mechanism of ZBP1 in impairing tumor growth in vivo, the SCC-7 tumor-bearing mice with normal and overexpressed ZBP1 were collected for single-cell sequencing. The data analysis revealed distinct immune cell compositions through clustering into 21 annotated cell types (**Fig 6A and 6B**). Five major immune populations were identified in **Fig 6C** based on canonical markers: CD4^+^ T cells (CD3D^+^, IL7R^+^), CD8^+^ T cells (CD3D^+^, CD8B^+^), NK cells (KLRF1^+^), B cells (MS4A1^+^), and Tregs (Foxp3^+^, CD25^+^). Comparative analysis demonstrated significant differential abundance of CD8^+^ T cells between experimental groups (**Fig 6D**). To further investigate the regulatory effects of ZBP1 overexpression on immune infiltration in SCC-7 mouse subcutaneous tumor tissues, we used flow cytometry to detect the infiltration of tumor-associated macrophages in SCC-7 mouse tumor tissues. Flow cytometric analysis of dissociated tumors revealed elevated CD8^+^ T cells infiltration and increased proportions of activated CD8^+^IFN-γ^+^ T cells in ZBP1-OE groups relative to controls (**Fig 6E and 6F**), with significant differences (**Fig 6H and 6I**). No significant differences were observed in CD8^+^PD-1^+^ (exhausted) or CD4^+^ T cell populations between groups (**S9** **and** **S10 Figs**). However, compared with control mice, the proportion of M2-type macrophages (CD11b^+^F4/80^+^CD206^+^, gating strategy shown in **S11 Fig**) in the subcutaneous tumor microenvironment of ZBP1-overexpressing mice was significantly reduced (**Fig 6G** and **6J**). Therefore, we used flow cytometry to detect the proportion and activation level of T cells in the spleens of SCC-7 subcutaneous tumor model mice (**S12A Fig**). Systemic immune activation was evidenced by increased splenic CD8^+^IFN-γ^+^ T cell frequencies in ZBP1-OE mice (*p* < 0.0001; **S12****B-****S12C Fig**), despite comparable total splenic CD8^+^ T cells counts (**S12****D-****S12E Fig**). These results demonstrated the promoting role of ZBP1 in CD8^+^ T cells infiltration in HNSCC tumor.

### 2.7. ZBP1 Enhances CD8^+^ T cells recruitment and activation via chemotactic signaling

**Fig 7:**
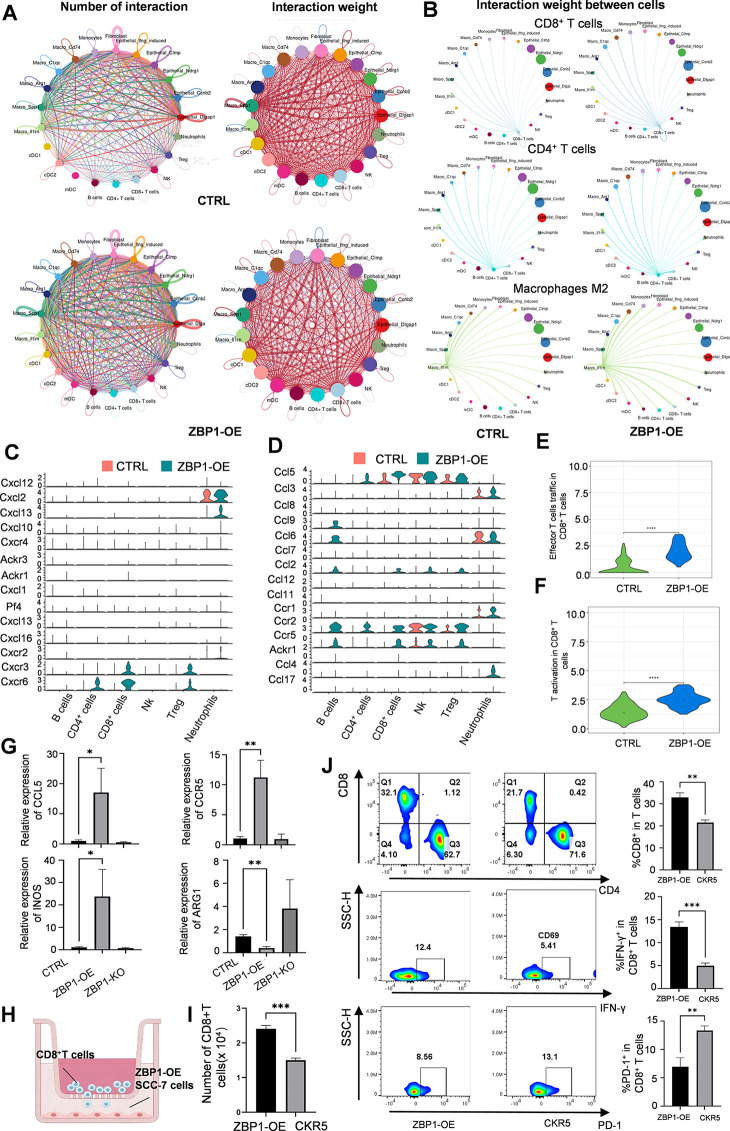
ZBP1 orchestrates the CCL5-CCR5 chemotactic axis to drive T cell recruitment and myeloid reprogramming. **(A–B)** Intercellular communication landscape in HNSCC tumors. **(A)** Circle plots illustrating the global increase in ligand-receptor interaction weights and numbers in ZBP1-OE compared to control tumors. **(B)** Cell-centric communication networks highlighting amplified crosstalk between CD8^+^ T cells, CD4^+^ T cells, and macrophages in the ZBP1-OE microenvironment. **(C–D)** Single-cell transcriptomic profiling of chemoattractant signaling. Violin plots show the specific upregulation of chemotactic receptors (Cxcr3, Cxcr6, Ccr2, Ccr5) and their dominant ligand (Ccl5) in ZBP1-OE infiltrating CD8^+^ T cells. **(E–F)** Flow cytometric quantification of T cell subsets *in vivo*. ZBP1-OE tumors exhibit a significant expansion of effector (**E**) and activated **(F)** CD8^+^ T cell populations (**** *p* < 0.0001). **(G)** Bidirectional molecular validation. qPCR analysis demonstrates that ZBP1 overexpression induces, while ZBP1 knockout (KO) suppresses, the expression of chemotactic transcripts (CCL5, CCR5) and M1-polarization markers (INOS) while negatively regulating the M2 marker Arg1. **(H-J)** Functional validation of the ZBP1-dependent CCL5-CCR5 axis using primary CD8^+^ T cells. (**H-I**) Schematic and statistical analysis of Transwell migration assays. The ZBP1-OE induced chemoattraction of T cells was significantly abolished by the CCR5 inhibitor (CKR5), demonstrating the functional requirement of this axis. **(J)** Characterization of T cell state in co-culture (n = 3). ZBP1-OE tumor cells significantly increased total CD8^+^ T cell counts and enhanced effector function (IFN-γ^+^) without driving terminal exhaustion (PD-1^+^), Statistical notation: Student-t test, (**p < 0.01; ***p < 0.001).

To elucidate the mechanisms driving ZBP1-dependent CD8^+^ T cell recruitment, we integrated cell-cell communication analysis with spatial transcriptomic profiling. ZBP1-OE tumors exhibited enhanced ligand-receptor crosstalk, with a 1.65-fold increase in interaction numbers (*p* < 0.001) and 1.44-fold stronger interaction weights (*p* < 0.01; **Figs 7A and** **S13****A**) compared to controls. Subgroup analysis revealed amplified communication between CD8^+^ T cells, CD4^+^ T cells, and macrophages, suggesting a coordinated immunoregulatory network (**Figs 7B** **and S13B**).

Spatial transcriptomic profiling revealed that ZBP1-ZBP1-OE CD8^+^ T cells exhibited marked upregulation of chemotactic receptors (CXCR3, CXCR6, CCR2, CCR5) and the ligand CCL5, positioning these cells as central mediators of immune cell recruitment (**Fig 7C and 7D**). Complementary pathway analysis further identified a multimodal signaling network in ZBP1-OE CD8^+^ T cells, characterized by enhanced incoming signals via immunoregulatory axes such as ADGRE5-CD55, SIRPα-CD47, and ICAM1-ITGAL, alongside cholesterol metabolism-related interactions (e.g., LAIR1-collagen, NECTIN3-TIGIT). Conversely, outgoing signaling was dominated by T cell activation and effector functions, including MHC-I-mediated antigen presentation (e.g., HLA-A/B/C-β2M), adhesion reinforcement (SELPLG-CD34, VCAM1-ITGAL), and cytotoxic priming through TRAIL-TRAILR and FASLG-FAS interactions (**S14 Fig**). Key pathways driving CD8^+^ T cell recruitment and activation included the CCL5-CCR5 chemotaxis axis signaling (*p* < 0.001). Functional validation confirmed these findings: ZBP1-OE tumors harbored 1.9-fold more effector CD8^+^ T cells (*p* < 0.0001; **Fig 7E**) and elevated activated subsets (*p* < 0.0001; **Fig 7F**). Collectively, ZBP1 orchestrates CD8^+^ T cell recruitment and cytotoxicity by synchronizing chemokine signaling (CXCR3/CCR5-CCL5), adhesion molecule interplay (SELPLG/CD48), and death receptor activation (TRAIL/FASLG), establishing it as a multimodal regulator of antitumor immunity.

To validate these mechanistic predictions, we performed molecular and functional perturbation assays. qPCR confirmed that ZBP1 overexpression significantly upregulated CCL5, CCR5, and the M1 marker iNOS while suppressing the M2 marker Arg1; conversely, ZBP1-KO led to opposite effects (**Fig 7G**). Consistent with the upregulation of Ccl5 and Ccr5 mRNA levels observed via qPCR, the CCR5 inhibitor (CKR5) effectively abolished the ZBP1-induced recruitment of primary CD8^+^ T cells in Transwell assays (**Fig 7H and 7I**). Flow cytometric analysis further demonstrated that ZBP1 not only increased T cell infiltration but also enhanced their effector function (IFN-γ^+^) without driving terminal exhaustion (PD-1^+^) (**Fig 7J**).

## 3. Discussion

Our integrative multi-omics strategy identified ZBP1 as a central orchestrator of HNSCC immunogenicity through three distinct lines of evidence. First, ZBP1 emerged as the top hub gene correlating with cytotoxic immune signatures, surpassing other differentially expressed genes in predictive power. Second, pan-cancer analysis revealed tumor-specific ZBP1 overexpression in HNSCC compared to 18 malignancies, contrasting its tumor-suppressive roles in melanoma through divergent immune modulation mechanisms [35]. Third, spatial co-localization with adaptive (CD8^+^/CD4^+^ T cells) and innate (CD68^+^ macrophages) immune infiltrates underscores its broader regulatory scope, which was potentially mediated by CCL5-CCR5 signaling activation.

HNSCC remains a therapeutically challenging malignancy, with limited advancements despite the clinical application of ICIs. While biomarkers such as PD-L1 and tumor mutational burden have been investigated, the functional relevance of nucleic acid sensors like ZBP1 in shaping the HNSCC TIME is poorly characterized. Although ZBP1 has been implicated in immunogenic cell death via RIPK3-MLKL-mediated necroptosis and shown to enhance antitumor immunity through dendritic cell and cytotoxic T lymphocyte recruitment in melanoma and colorectal cancer [34,38,39], its specific role in regulating CD8^+^ T cell dynamics and TIME remodeling in HNSCC remains undefined. Our study addresses this knowledge gap by systematically integrating multi-omics profiling and functional assays to establish ZBP1 as a central regulator of immune-mediated tumor control in HNSCC.

The prognostic significance of immune-related genes in HNSCC has been extensively documented, with CXCL13, CD103, and PD-L1 serving as established markers of immune infiltration and survival [40]. There are also new genes such as immune cell infiltration-associated ADME, OLR1 and SPP1^+^ that have been developed and validated in HNSCC [41–44]. However, ZBP1’s association with clinical outcomes in HNSCC had not been systematically investigated. Leveraging TCGA-HNSCC datasets, we identified ZBP1 as a novel prognostic biomarker, with high expression correlating with improved overall survival and reduced disease-specific mortality. Notably, ZBP1 expression inversely correlated with advanced tumor stage (T4/Stage IV), suggesting its progressive loss may drive immune evasion during tumor evolution. These findings align with reports in colorectal cancer [34,38,39], where ZBP1 deficiency accelerates tumor progression by suppressing necroptosis-driven immune activation [17]. Our immunohistochemical validation further confirmed ZBP1’s spatial co-localization with CD8^+^ T cells and macrophages in clinical specimens, underscoring its immunomodulatory relevance.

The biological function of ZBP1 in cancer appears to be highly context-dependent and tissue-specific. Prior studies in melanoma and colorectal cancer have primarily positioned ZBP1 as a mediator of immunogenic cell death, specifically through RIPK3-MLKL-dependent necroptosis, which subsequently triggers the cGAS-STING pathway via mitochondrial DNA release to prime systemic immunity. While these studies emphasize ZBP1 as a ‘death switch,’ our findings in HNSCC uncover a distinct, multifaceted regulatory network. Unlike the predominantly necroptosis-centric mechanism observed in melanoma, ZBP1 in the HNSCC microenvironment functions as a master coordinator of chemotaxis and myeloid reprogramming. We observed that ZBP1 directly orchestrates the CCL5-CCR5 signaling axis—a pathway not previously highlighted as the primary driver in melanoma ZBP1 models—to actively recruit CD8^+^ T cells into the tumor nest. Furthermore, our study reveals a novel role for ZBP1 in reversing the immunosuppressive myeloid landscape by shifting macrophages from an M2-like to an M1-like phenotype, a mechanism that appears particularly critical given the highly ‘cold’ and myeloid-dense nature of HNSCC. This divergence suggests that ZBP1’s anti-tumor efficacy is not limited to inducing cell death but extends to a proactive remodeling of the immune architecture, making it a unique therapeutic target for ‘cold’ squamous cell carcinomas.

To delineate ZBP1’s functional impact on tumor growth, we established ZBP1-OE subcutaneous tumor models in SCC-7 and MOC2 cells. ZBP1-OE tumors exhibited significantly attenuated growth compared to controls, a phenomenon attributed not only to intrinsic tumor suppression but also to enhanced immune infiltration. This dual mechanism mirrors previous findings in melanoma, where ZBP1-driven necroptosis promotes mitochondrial DNA (mtDNA) [10,38]. Importantly, ZBP1’s tumor-suppressive effects in HNSCC appear context-dependent: while ZBP1 knockout did not alter in vitro proliferation, its in vivo overexpression reshaped the TIME by reducing M2 macrophage polarization and amplifying CD8^+^ T cell activation. Such immune-mediated suppression aligns with recent reports highlighting ZBP1’s role in radiation-induced abscopal effects, where mtDNA-cGAS-STING signaling primes systemic antitumor immunity [38].

The recruitment and activation of CD8^+^ T cells are pivotal for effective antitumor immunity, yet the molecular drivers of this process in HNSCC remain elusive. Our study reveals ZBP1 as a multimodal regulator of T cell recruitment, operating through chemokine signaling (CCL5-CCR5 axis), adhesion molecule interactions (SELPLG-CD34), and cytotoxic priming (TRAIL/FASLG). Single-cell RNA sequencing of ZBP1-OE tumors uncovered upregulated chemotactic receptors (CXCR3, CCR5) in CD8^+^ T cells, facilitating their infiltration into tumor nests. These findings resonate with recent discoveries in leukemia models, where CD8^+^ T cells exhibit organ-specific trafficking via integrin-mediated adhesion (e.g., ITGB7-MAdCAM-1) [45]. Furthermore, spatial transcriptomics highlighted ZBP1’s role in amplifying crosstalk between CD8^+^ T cells and antigen-presenting cells (APCs), a mechanism paralleling the MHC-Ib-restricted CD8^+^ T cell activation reported in hepatocellular carcinoma [46]. Despite these advances, our mechanistic understanding of ZBP1 remains incomplete. While we identified key pathways (e.g., CCL5-CCR5) driving T cell recruitment, the direct targets of ZBP1 in regulating CCL-CCR5 signaling was still unclear. Furthermore, the upstream triggers of ZBP1 activation—whether radiation-induced DNA damage, viral nucleic acids, or endogenous retroelements—require further investigation. Notably, ADAR1, an RNA-editing enzyme that masks immunogenic dsRNA, has been shown to suppress ZBP1 activation in melanoma, suggesting a potential therapeutic axis for reversing immune resistance in HNSCC [29,34]. Additionally, the interplay between ZBP1 and metabolic regulators like PYGL, which fosters lactate-mediated immunosuppression, warrants exploration to optimize combinatorial therapies [45].

In conclusion, our study establishes ZBP1 as a pivotal regulator of CD8^+^ T cell-mediated immunity in HNSCC, operating within a complex network of chemotactic, adhesive, and cytotoxic signals. Its dual role in tumor suppression and immune activation positions ZBP1 as a promising therapeutic target, particularly when combined with radiotherapy or ICIs strategies for HNSCC, offering a roadmap to overcome immunosuppression and improve clinical outcomes.

## 4. Methods

### 4.1. Ethics statement

Ethical approval for the collection of human data was obtained from the Institutional Review Board (No.2025314) of West China Hospital. Prior to enrollment, all participants provided written informed consent. Animal Management Regulations and were ethically approved by West China Hospital’s the Institutional Animal Care and Use Committee (IACUC) (No.20250418002).

### 4.2. Clinical specimens for experiments

Clinicopathological specimens were obtained from the Department of Pathology at West China Hospital, Sichuan University, with approval from the Biomedical Ethics Review Committee of West China Hospital of Sichuan University and informed consent was obtained from all patients. These specimens comprised surgically resected samples from treatment-naïve patients diagnosed with primary HNSCC between 2015 and 2019.

### 4.3. Bioinformatic analysis

We analyzed transcriptomic profiles from 504 primary HNSCC tumors and 44 matched adjacent normal tissues obtained from The Cancer Genome Atlas (TCGA) portal. Differential gene expression analysis was conducted using the Limma package (v3.52.4) in R. To ensure a standardized and robust stratification approach across analyses, different thresholding criteria were applied based on the analysis objective. For the initial identification of genes associated with immune infiltration, samples were stratified into high and low groups based on the median CD8^+^ T cell infiltration score. For the subsequent identification of ZBP1-related DEGs and all survival analyses (Overall Survival and Disease-Specific Survival), we utilized quartile splitting, defining the ‘high’ group as the top 25% and the ‘low’ group as the bottom 25% of ZBP1 expression levels. This quartile-based approach was chosen to maximize the biological contrast between the groups and improve the sensitivity of the prognostic model. Prior to analysis, low-abundance genes (CPM < 1) were filtered, and TMM normalization was applied. For TCGA-HNSCC datasets, the ComBat function was utilized to mitigate potential batch effects across different sequencing runs. By systematically integrating clinical annotations, we compared transcriptomes between tumor and normal tissues to identify differentially expressed genes (DEGs), while ensuring cohort alignment with established HNSCC molecular subtypes; then, by using the TIMER algorithm in the IOBR package of R software, CD8^+^ T cell infiltration levels in HNSCC tissues were quantified, followed by comparative transcriptomic analysis between high- versus low-infiltration cohorts using the Limma package to identify DEGs [47]. On this basis, using the Sangerbox 3.0 platform (http://sangerbox.com), we systematically evaluated ZBP1 expression across 25 HNSCC tumors and paired adjacent normal tissues, followed by correlation analysis between ZBP1 levels and tumor immune infiltration scores using CIBERSORTx-derived lymphocyte quantification [48]. Survival analysis of ZBP1 expression and prognosis of HNSCC patients as well as correlation analysis of ZBP1 expression and HNSCC tumor stage were also performed. Finally, using the Researcher’s House platform (https://www.home-for-researchers.com), HNSCC specimens were stratified into ZBP1-high and ZBP1-low groups based on median expression thresholds. Differential gene expression analysis between these cohorts identified 427 significantly dysregulated transcripts (|log2FC| > 1, FDR < 0.05), which subsequently underwent KEGG/GO pathway enrichment analyses [49]. Parallelly, GSVA-based deconvolution of TCGA-HNSCC transcriptomes quantified CD8^+^ T cell activation signatures [41], revealing spatial correlation patterns with ZBP1 expression gradients [50].

### 4.4. Cell lines and cell culture

The SCC-7 (RRID: CVCL_V412) and MOC2 (RRID: CVCL_ZD33) cell lines, along with human FaDu (RRID: CVCL_1218) and FD-LSC-1 (RRID: CVCL_S891) cells, were obtained from the State Key Laboratory of Biotherapy, West China Hospital, Sichuan University. All cell lines were cultured at 37°C in a 5% CO2 humidified incubator using RPMI-1640 (SCC-7) or DMEM (MOC2, FaDu, FD-LSC-1) medium supplemented with 10% fetal bovine serum (ZATA, #Z7185FBS-500) and 1% penicillin-streptomycin (Gibco). All cell lines were authenticated using STR profiling within the last three years (S1-S3 Text files). All experiments were performed with mycoplasma-free cells as confirmed by regular testing using the MycoAler Mycoplasma Detection Kit (Lonza). Mycoplasma contamination was routinely monitored throughout the experiments.

### 4.5. Cell proliferation assay1

Cellular proliferation was quantified using complementary CCK-8 and clonogenic assays. SCC-7 cells were seeded in 96-well plates (5 × 10^3^ cells/well) and exposed to 10% CCK-8 solution at 24, 48, and 72-hour intervals, with subsequent absorbance quantification at 450 nm. Parallel clonogenic potential was assessed by culturing cells in 6-well plates (2 × 10^3^ cells/well) for 14 days, followed by PBS washing, 4% paraformaldehyde fixation, and crystal violet staining (Solarbio, China).

### 4.6. Western blot analysis

Cellular proteins were isolated using a commercially available lysis reagent (Beyotime, China) and quantified through bicinchoninic acid (BCA) assay with standardized calibration curves. Equal protein aliquots were resolved by SDS-PAGE (Epizyme Biotech, China) and transferred to PVDF/nitrocellulose membranes. After blocking (Epizyme Biotech), membranes were probed with primary antibodies (4°C overnight) and secondary antibodies (1 h at room temperature). Antibody details are provided in S1 Table.

### 4.7. Flow cytometry assay

Single-cell suspensions of SCC-7 cells were generated through enzymatic dissociation with 70-μm filtration to ensure monodispersity. Apoptotic dynamics were quantified via Annexin V-APC/PI dual staining (Elabscience) under standardized conditions: cells in binding buffer were sequentially incubated with Annexin V-APC (20 min, 4°C) and PI (5 min, RT), followed by immediate acquisition on a BD FACS Canto II. FlowJo (v10.8.1; BD Biosciences) was employed to gate four distinct populations: viable (Annexin V ^⁻^ /PI^⁻^), early apoptotic (Annexin V ^+^ /PI^-^), late apoptotic (Annexin V ^+^ /PI^+^), and necrotic (Annexin V ^-^ /PI^+^) cells using quadrant-based fluorescence thresholds.

All cellular assays, including CCK-8, clonogenic, and Western blot, were performed in triplicate (n = 3 independent biological replicates).

### 4.8. Animal models

Female C3H/HeJ mice (5–6 weeks old) were obtained from SPF Biotechnology (Beijing) and housed under specific pathogen-free (SPF) conditions (22°C, 50% humidity, 12h light/dark cycles) with ad libitum access to food/water. All procedures complied with China’s Laboratory Animal Management Regulations and were approved by the Institutional Animal Care and Use Committee (IACUC) of West China Hospital (No. 20250418002). Tumor Model and Monitoring: Subcutaneous tumors were established via SCC-7/MOC2 cell inoculation. Mice were assigned to experimental groups using a computer-generated random number generator to ensure unbiased allocation. To maintain experimental rigor and ensure blinding, tumor measurements and endpoint assessments were performed by two independent investigators who were unaware of the specific treatment assignments. The sample size (n = 6 per group) was determined based on a power calculation (β = 0.8, α = 0.05) derived from our preliminary experiments, aiming to detect at least a 30% difference in tumor volume between the control and treatment groups. Tumor dimensions were measured triweekly with digital calipers, and volumes calculated as (length × width^2^)/2. Body weight and clinical scores were recorded to assess systemic health. Animals were immediately euthanized via CO_2_ inhalation (gradual displacement, 30% chamber volume/min) if tumors exceeded 2000 mm^3^, ulcerated, or if body weight loss surpassed 20%. No animals reached these thresholds during the study. Investigators were blinded to group assignments during data collection/analysis.

### 4.9. Immunohistochemistry (IHC)

Formalin-fixed tumor specimens were sectioned into 4-μm slices followed by deparaffinization and graded ethanol rehydration. Microwave-mediated antigen retrieval was performed using citrate/EDTA buffer (pH 6.0/9.0). After quenching endogenous peroxidases with 3% H_2_O_2_ and blocking with 10% goat serum, sections were incubated with primary antibodies (Proteintech): anti-CD8 (#66868–1-Ig; 1:400), anti-CD4 (#67786–1-Ig; 1:400), and anti-ZBP1 (#84396–2-RR; 1:200) overnight at 4°C. Signal amplification employed sequential biotinylated IgG and streptavidin-HRP (20 min each), with DAB chromogen (Mxb Biotechnologies) visualization and hematoxylin nuclear counterstaining.

### 4.10. Immunofluorescence staining

Following fixation with 4% paraformaldehyde and permeabilization with 0.2% Triton X-100, cells were blocked with 5% BSA (1 h, RT). Primary antibodies (Proteintech) targeting immune markers were incubated overnight at 4°C at 1:400 dilutions: anti-ZBP1, anti-CD8, anti-CD4, anti-CD11b (#66519–1-Ig), and anti-CD68 (#66231–2-Ig). After PBS washes, fluorophore-conjugated secondary antibodies (Abbkine) were applied (1 h, RT), followed by DAPI nuclear counterstaining (Beyotime). Confocal imaging was performed using an OLYMPUS system with FV10-ASW Viewer software for z-stack analysis.

### 4.11. Molecular biology methods

Plasmid DNA was purified from E. coli using the TIANGEN Endo-Free Plasmid Midi Kit. Bacterial pellets were lysed with Buffers P1/P2, neutralized with Buffer P4, and processed via CP4 columns with isopropanol precipitation. Purified DNA was eluted in TE buffer (pH 8.0) after sequential washes with PD and PW buffers. DH5α competent cells were transformed with 50–100 ng plasmid DNA by heat shock (42°C, 60 s), plated on LB agar containing 100 μg/mL ampicillin, and incubated overnight at 37°C. Selected colonies were cultured in LB medium for plasmid amplification.

Genomic DNA extraction was performed using the TIANGEN Genomic DNA Kit following manufacturer’s protocol. Cellular lysis was achieved through incubation with Buffer GA/GB containing 20 mg/mL Proteinase K at 56°C for 4 hr. Subsequent DNA purification involved ethanol precipitation followed by binding to GD columns. After two washing steps with Buffer PW, purified DNA was eluted in TE buffer (10 mM Tris-HCl, 1 mM EDTA, pH 8.0) and quantified by spectrophotometry.

PCR amplification was conducted in 10-μL reaction volumes containing 100 ng template DNA, 0.5 μM forward/reverse primers, and 0.5 U Phanta Super-Fidelity DNA Polymerase (Vazyme). Thermal cycling parameters consisted of an initial denaturation at 95°C for 2 min, followed by 35 cycles of denaturation (95°C, 10 s), primer annealing (58°C, 10 s), and extension (72°C, 30 s), with a final extension step at 72°C for 7 min.

For T7 Endonuclease I (T7EI) assays, 5 μL PCR products were denatured/annealed (95°C to 16°C gradient) in 1 × T7 buffer, then incubated with 0.2 μL T7EI at 37°C for 30 min. Digested fragments were resolved on 2% agarose gels (TAE buffer, 100 V, 40 min) pre-stained with GelRed. Electrophoresis was performed using 2% agarose gels containing GelRed (1:10,000), with DNA samples mixed in 6 × loading dye and separated at 100 V for 40 min.

### 4.12. Single-cell RNA sequencing analysis

**Sample Preparation and Library Construction:** Tumor specimens from MOC-2 subcutaneous xenografts (ZBP1-overexpressing vs. Control) were enzymatically dissociated into single-cell suspensions. Following viability assessment (>85% by Trypan Blue exclusion), a targeted cell recovery of 8,000 cells per sample was achieved using the 10x Genomics Chromium platform (GemCode Single-cell Instrument). Single-cell libraries were constructed using the Chromium Single Cell 3′ Reagent Kit (v3.1) according to the manufacturer’s protocol. In brief, cellular suspensions were loaded onto microfluidic chips for barcoded bead encapsulation, followed by reverse transcription and cDNA amplification. Final libraries were quality-controlled via fragment size analysis and quantified by qPCR prior to sequencing on an Illumina NovaSeq 6000 platform with a targeted depth of 50,000 reads per cell.

**Data Preprocessing and Quality Control:** Raw sequencing data were processed using CellRanger (v6.0) for read alignment and cell calling. Downstream analysis was performed using the Seurat computational framework (v5.3) [ 51,52]. Rigorous quality control (QC) measures were applied: 1) cells were retained if they detected between 200 and 3,700 genes (calculated as twice the median value of 1,850) to ensure transcriptomic integrity; 2) cells with mitochondrial gene content exceeding 15% were excluded. To ensure each barcode represented a single cell, DoubletFinder was utilized for the systematic identification and removal of potential doublets.

**Normalization, Integration, and Clustering:** Data were normalized and scaled using the SCTransform workflow to stabilize variance. To mitigate batch effects between the control and ZBP1-OE samples while preserving biological heterogeneity, the Harmony package (v1.0) was utilized for data integration. Dimensionality reduction was performed via Principal Component Analysis (PCA) using the top 30 principal components. Based on a resolution gradient analysis (0.2–1.2) using clustree, an optimal clustering resolution of 0.5 was selected. Cells were visualized using Uniform Manifold Approximation and Projection (UMAP) and annotated based on canonical lineage markers, including epithelial (EpCAM^+^), stromal (VIM^+^), and immune (CD45^+^) populations.

**Intercellular Communication Analysis:** Cell-cell signaling interactions were quantified using CellChat (v2.0). We employed the mouse CellChatDB repository to evaluate ligand-receptor crosstalk between T cell subsets and macrophages. The rankNet framework was applied to compute normalized pathway activation scores (0–1 scale) across cellular subpopulations. Subtype-specific signaling patterns and differential‌‌ activation were illustrated using bubble plots (netVisual_bubble) and comparative bar plots.

### 4.13. Functional validation of mechanisms

Primary CD8^+^ T cells were isolated and co-cultured with SCC-7 cells. For rescue and interference assays, recombinant CCL5 (50 ng/mL) or the CCR5 inhibitor CKR5 (10 μM) was added to the co-culture system for 24 h. Migration was assessed using 8-μm Transwell inserts, and cell counts were quantified in five random fields. Myeloid polarization and T cell activation markers were analyzed via flow cytometry and Western blot as previously described.

### 4.14. Statistical analysis

**Statistical analyses were performed using GraphPad Prism (v9.0) and R software (v4.2.0). Data are presented as mean ± SEM from at least three independent biological replicates. For comparisons between two groups, two-tailed Student’s t-tests were employed. For multiple-group comparisons, one-way ANOVA followed by Tukey’s post-hoc test was applied. P-values < 0.05 were considered statistically significant (*p < 0.05, **p < 0.01, ***p < 0.001, ****p < 0.0001).

## Supporting information

S1 Fig Differential expression of ZBP1 in HNSCC. (A) Pan-cancer analysis of ZBP1 expression between tumor and adjacent normal tissues in TCGA database (*p < 0.05, **p < 0.01, ***p < 0.001, ****p < 0.0001, paired t-test). (B) Representative immunohistochemical (IHC) staining of ZBP1 in HNSCC tissues.Scale bars: 50 μm. (C) Quantitative analysis of ZBP1 expression in tumor versus adjacent tissues using IHC scoring (sum of staining intensity [0–4] and positive cell proportion [0–4]). Statistical significance determined by unpaired t-test (****p < 0.0001).
(TIF)

S2 Fig ZBP1 overexpression in both SCC-7 cells. (PNG)

S3 Fig ZBP1 overexpression in both MOC2 cells. (PNG)

S4 Fig ZBP1 overexpression promotes cellular apoptosis. (A) Flow cytometric analysis of apoptotic proportions in SCC-7-CTRL vs ZBP1-OE cells. (B) Quantification of early apoptotic cells (Annexin V ^+^ PI^−^) (C) Quantification of total apoptotic cells (Annexin V ^+^ PI^+^ plus Annexin V ^+^ PI^−^) (ZBP1-OE vs Ctrl: **** *p* < 0.0001, Student’s t-test, n = 3 biological replicates).
(TIF)

S5 Fig Generation and validation of ZBP1-knockout HNSCC cell lines. (A) CRISPR sgRNA sequences targeting murine ZBP1. (B) T7 endonuclease I assay verifying ZBP1 genome editing: PCR-amplified genomic fragments (upper) and cleaved products post-T7EI digestion (lower, arrows). (C) Sanger sequencing confirmation of frameshift mutations in ZBP1-knockout clones. (D) Radiation-induced ZBP1 transcriptional changes (RT-qPCR; ***p < 0.001 vs Ctrl). (E) ZBP1 protein expression post-irradiation (Western blot). (Statistical notation: *p < 0.05, ** *p* < 0.01, *** *p* < 0.001, **** *p* < 0.0001. Group labels: CTRL (empty vector), RT (radiation-treated), ZBP1-KO1/KO2 (knockout clones).).
(TIF)

S6 Fig Validation of ZBP1 knockout (ZBP1-KO) in SCC-7 cells by Western blot. (PNG)

S7 Fig ZBP1 knockout does not affect proliferation and clonogenicity in HNSCC cells. (A) CCK-8 assay showing comparable growth curves between KO-Ctrl and two ZBP1-KO monoclonal clones. (B) Representative colony formation assay demonstrating equivalent clonogenic capacity across groups. (C) Quantification of colony numbers (ns.: not significant, one-way ANOVA, n = 3 biological replicates). Group labels: CTRL (empty vector), RT (radiation-treated), ZBP1-KO1/KO2 (knockout clones).).
(TIF)

S8 Fig ZBP1 knockout does not influence apoptosis in HNSCC cells. (A) Flow cytometry analysis of apoptosis in SCC-7 cells (KO-Ctrl vs two ZBP1-KO clones). (B) Quantification of early apoptotic cells (Annexin V ^+^ PI^−^) (C) Quantification of total apoptotic cells (Annexin V + PI+ plus Annexin V ^+^ PI^−^) (No significant differences [n.s.] by one-way ANOVA, n = 3 independent experiments; Group labels: CTRL (empty vector), RT (radiation-treated), ZBP1-KO1/KO2 (knockout clones).).
(TIF)

S9 Fig T cell infiltration and activation profiling in murine tumor microenvironment. (A) Flow cytometry gating strategy for T cell detection in subcutaneous tumors. (B-C) CD8 ^+^ PD-1 ^+^ T cell infiltration (No significant differences [n.s.] by Student’s t-test). (D-E) CD4 ^+^ T cell infiltration (No significant differences [n.s.] by Student’s t-test). Group labels: Ctrl (control), OE(ZBP1-Overexpression).
(TIF)

S10 Fig Flow cytometry gating strategy for CD8 + T cell recruitment and functional characterization in co-culture assays. Representative gating strategy for identifying primary CD8 + T cells in the co-culture system.
(TIF)

S11 Fig Flow cytometric profiling of tumor-associated macrophages (TAMs) in murine tumor microenvironment. (A) Gating strategy for TAMs identification in subcutaneous tumor models. (B-C) CD11b^+^F480 ^+^cell proportions. (No significant differences [n.s.] by Student’s t-test, n = 3 independent experiments; Group labels: Ctrl (control), OE(ZBP1-Overexpression)).
(TIF)

S12 Fig Flow cytometric analysis of splenic T cell activation and infiltration in tumor-bearing mice. (A) Gating strategy for splenic T cell identification in subcutaneous tumor models. (B-C) IFN-γ ^+^ CD8 ^+^ T cell proportions within total CD8 ^+^ T cells (***p < 0.001 vs Ctrl, Student’s t-test). (D-E) CD8 ^+^ T cell frequency among CD3 ^+^ T cells (No significant differences [n.s.] by Student’s t-test. Group labels: Ctrl (control).
(TIF)

S13 Fig ZBP1 enhances intercellular communication networks in tumors. (A) Left panel: Quantitative comparison of ligand-receptor interaction numbers (bar plot, left) and interaction strength (bar plot, right) between control (CTRL) and ZBP1-overexpressing (ZBP1-OE) tumors. (B) Top panel: Heatmap of cell-cell interaction counts (left) and strength (right) across different subsets.
(TIF)

S14 Fig ZBP1-OE CD8 + T cells exhibit enriched signaling pathway engagement. (A) Incoming signaling patterns in ZBP1-OE vs. CTRL different subsets. Heatmap rows: incoming pathways; columns: cell clusters. Color scale: normalized enrichment scores (NES, Z-score). (B) Outgoing signaling patterns from ZBP1-OE different subsets. Heatmap rows: outgoing pathways; columns: target cell types. Color scale: Z-scored interaction strength.
(TIF)

S1 Table Reagents and resources used in this study. List of key reagents, including cell culture media, antibodies, assay kits, and critical chemicals, with corresponding manufacturers and countries of origin.
(DOCX)

S1 Text STR profiling of SCC-7 cell line. (PDF)

S2 Text STR profiling of Moc2 cell line. (PDF)

S3 Text STR profiling of Fadu cell line. (PDF)
