# Supplementary figures and images for: ZBP1 Drives CD8+ T cell-mediated anti-tumor immunity in head and neck squamous cell carcinoma
Source: PLoS Genet. 2026 May 26;22(5):e1012107. doi: 10.1371/journal.pgen.1012107 (PMC13249162; doi:10.1371/journal.pgen.1012107)

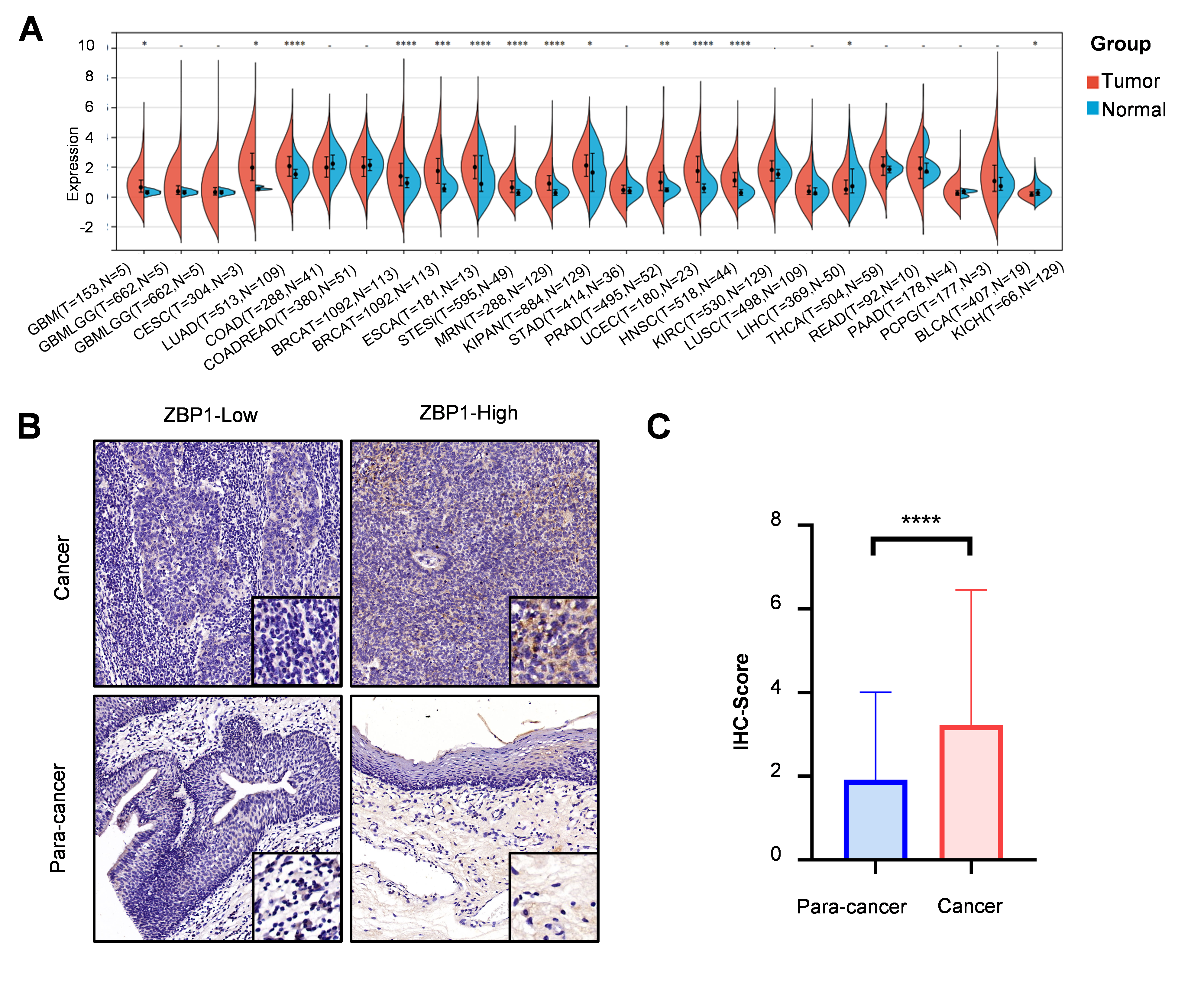

Supplement: S1 Fig — (A) Pan-cancer analysis of ZBP1 expression between tumor and adjacent normal tissues in TCGA database (*p < 0.05, **p < 0.01, ***p < 0.001, ****p < 0.0001, paired t-test). (B) Representative immunohistochemical (IHC) staining of ZBP1 in HNSCC tissues.Scale bars: 50 μm. (C) Quantitative analysis of ZBP1 expression in tumor versus adjacent tissues using IHC scoring (sum of staining intensity [0–4] and positive cell proportion [0–4]). Statistical significance determined by unpaired t-test (****p < 0.0001). (TIF) [file pgen.1012107.s001.tif]

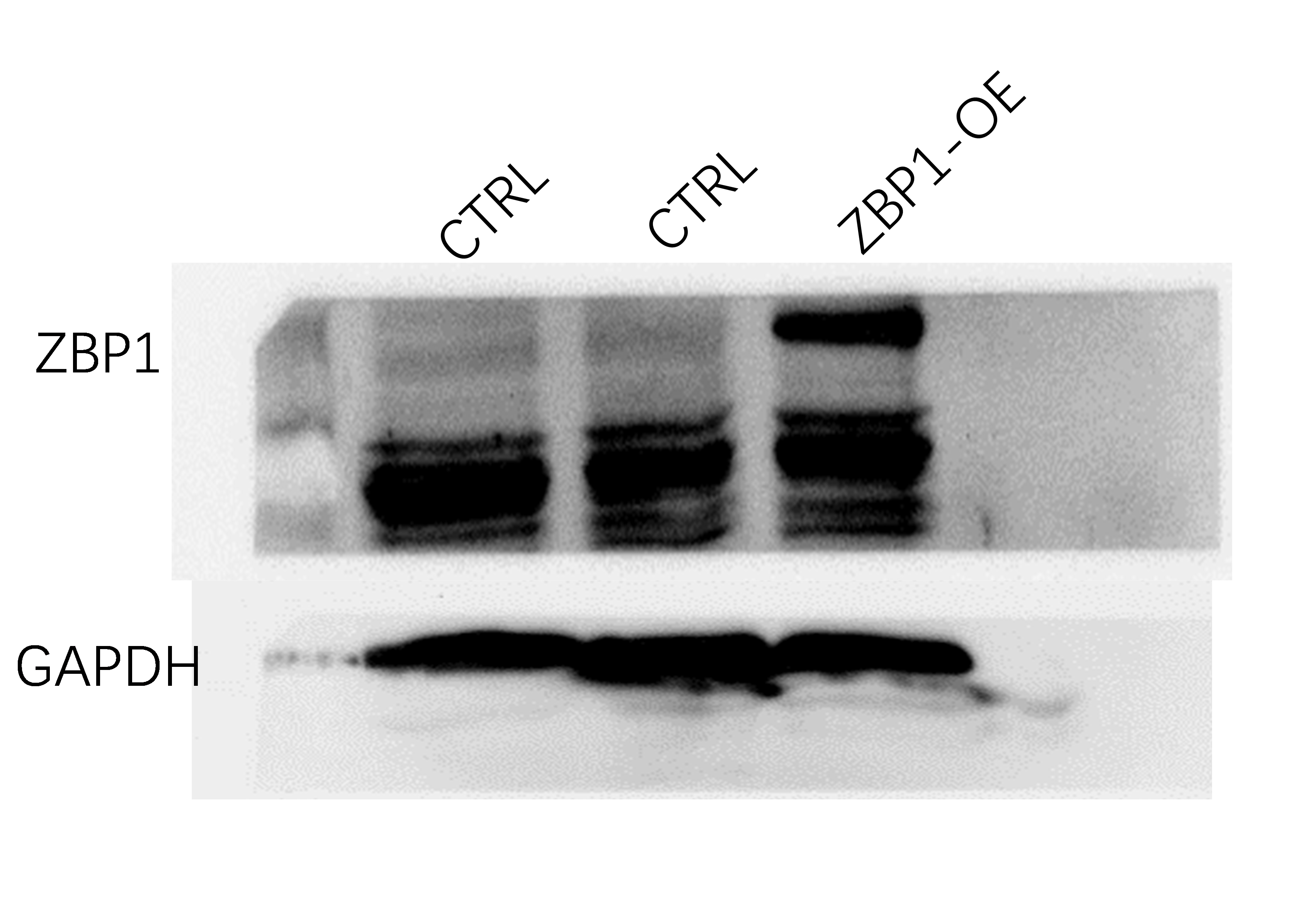

Supplement: S2 Fig — (PNG) [file pgen.1012107.s002.png]

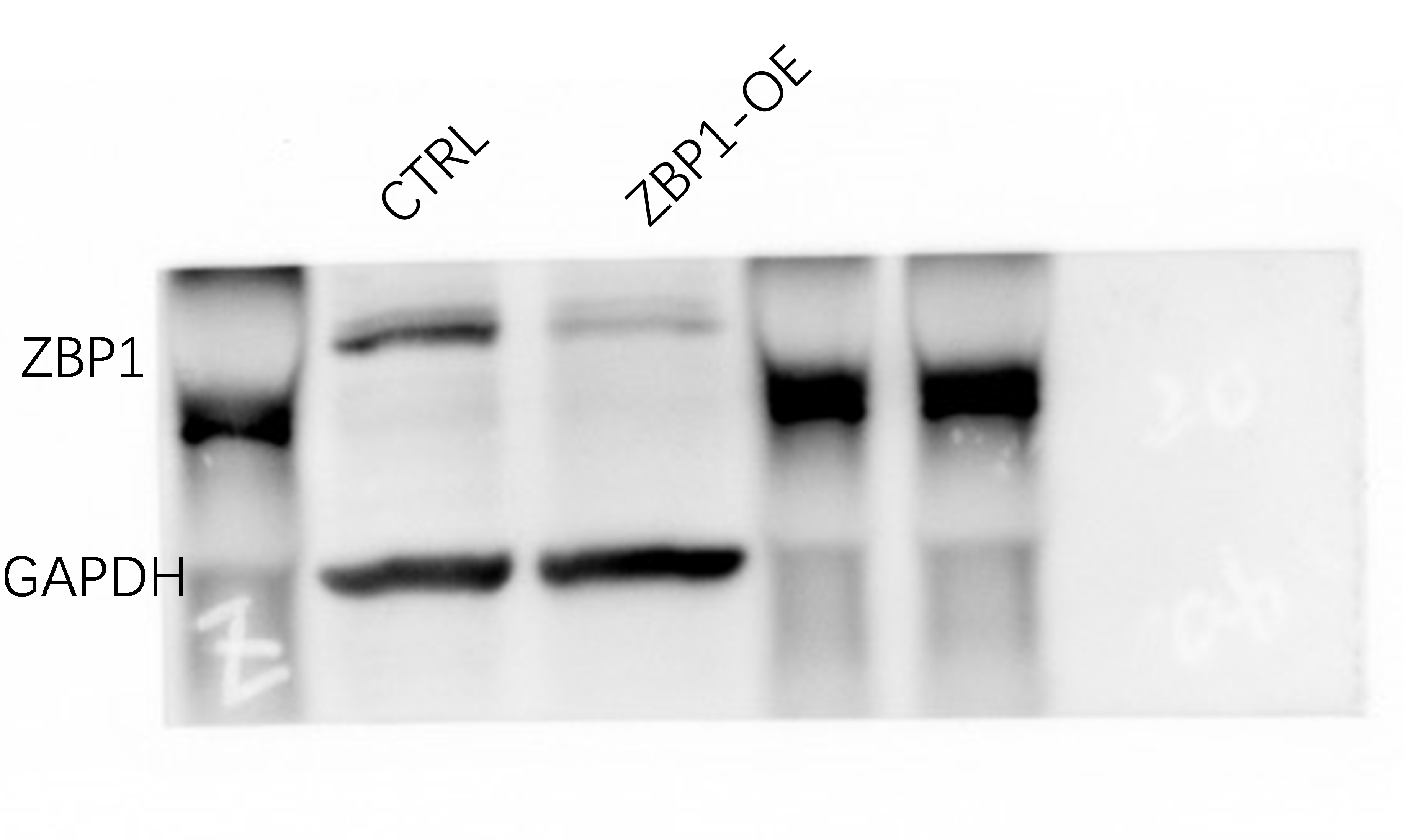

Supplement: S3 Fig — (PNG) [file pgen.1012107.s003.png]

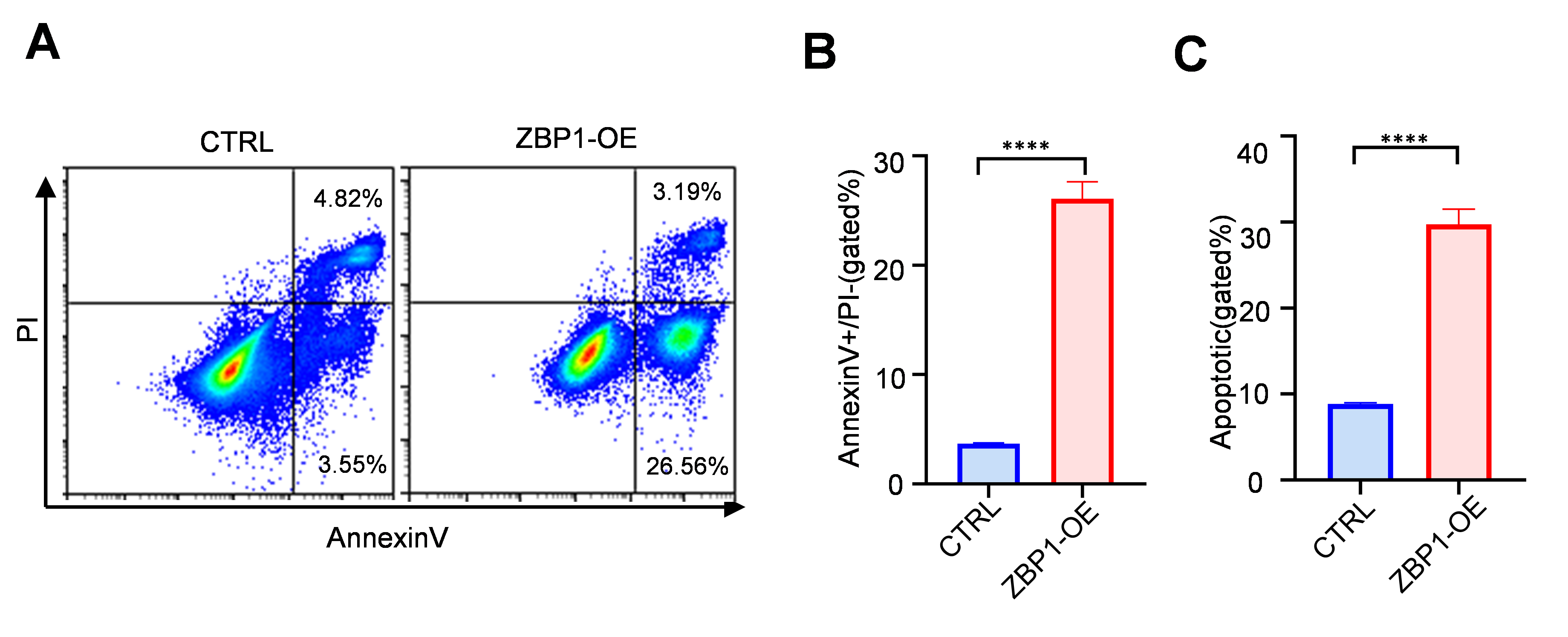

Supplement: S4 Fig — (A) Flow cytometric analysis of apoptotic proportions in SCC-7-CTRL vs ZBP1-OE cells. (B) Quantification of early apoptotic cells (Annexin V + PI−) (C) Quantification of total apoptotic cells (Annexin V + PI+ plus Annexin V + PI−) (ZBP1-OE vs Ctrl: **** p < 0.0001, Student’s t-test, n = 3 biological replicates). (TIF) [file pgen.1012107.s004.tif]

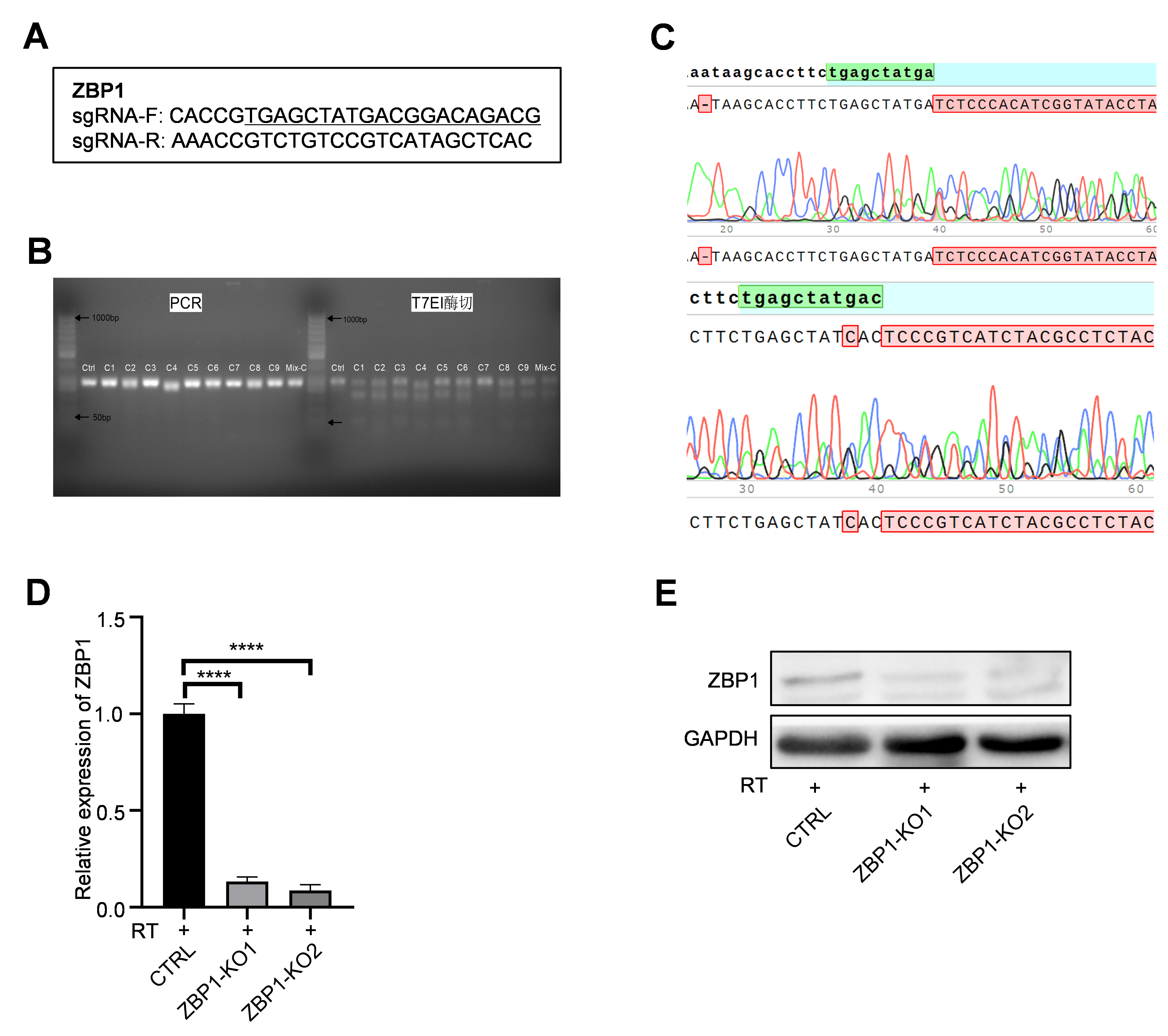

Supplement: S5 Fig — (A) CRISPR sgRNA sequences targeting murine ZBP1. (B) T7 endonuclease I assay verifying ZBP1 genome editing: PCR-amplified genomic fragments (upper) and cleaved products post-T7EI digestion (lower, arrows). (C) Sanger sequencing confirmation of frameshift mutations in ZBP1-knockout clones. (D) Radiation-induced ZBP1 transcriptional changes (RT-qPCR; ***p < 0.001 vs Ctrl). (E) ZBP1 protein expression post-irradiation (Western blot). (Statistical notation: *p < 0.05, ** p < 0.01, *** p < 0.001, **** p < 0.0001. Group labels: CTRL (empty vector), RT (radiation-treated), ZBP1-KO1/KO2 (knockout clones).). (TIF) [file pgen.1012107.s005.tif]

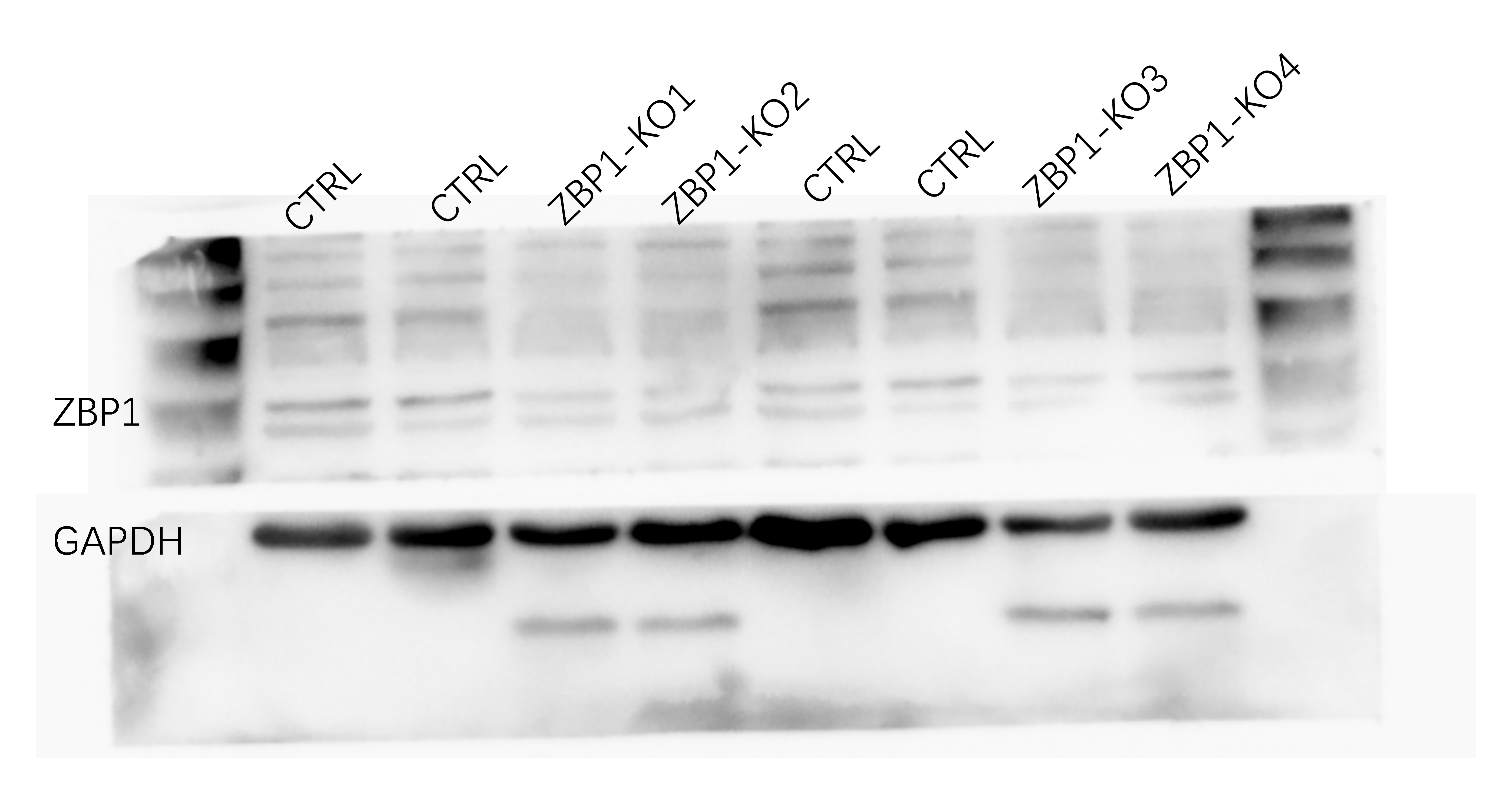

Supplement: S6 Fig — (PNG) [file pgen.1012107.s006.png]

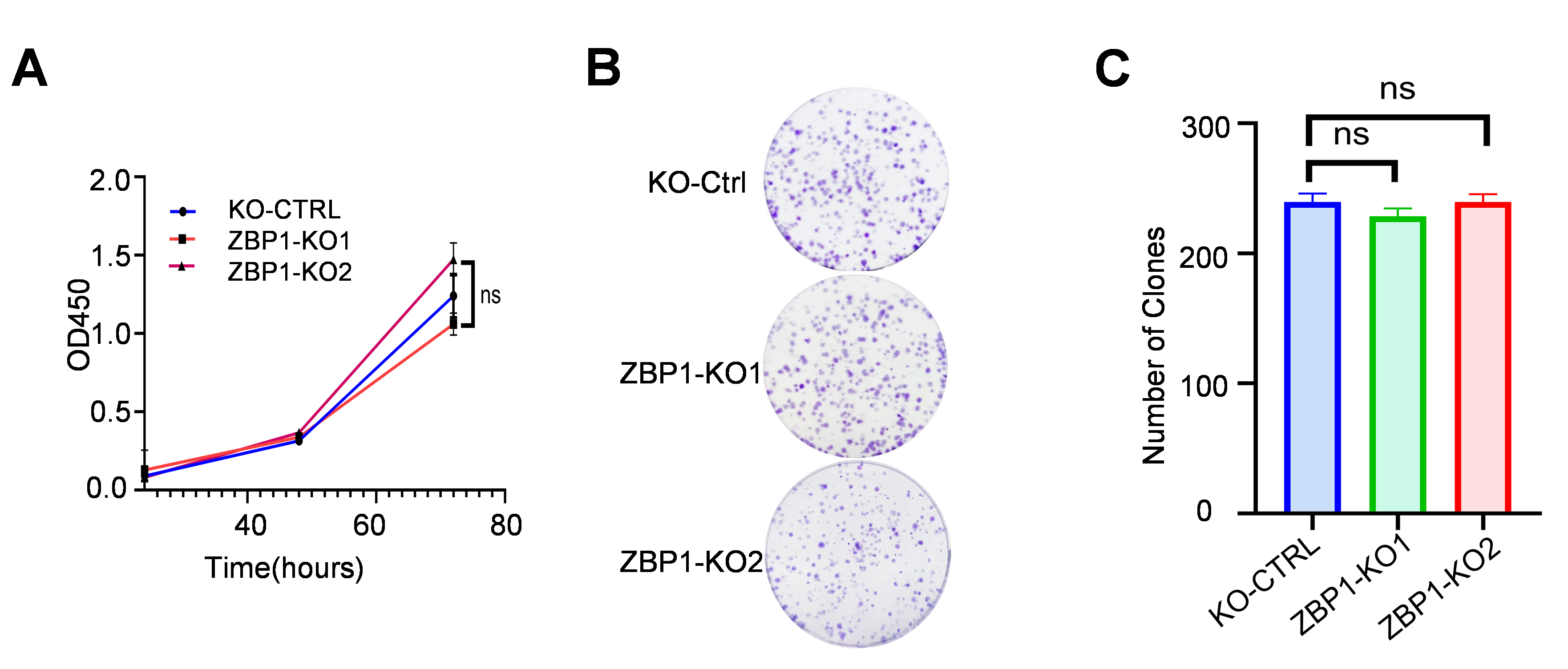

Supplement: S7 Fig — (A) CCK-8 assay showing comparable growth curves between KO-Ctrl and two ZBP1-KO monoclonal clones. (B) Representative colony formation assay demonstrating equivalent clonogenic capacity across groups. (C) Quantification of colony numbers (ns.: not significant, one-way ANOVA, n = 3 biological replicates). Group labels: CTRL (empty vector), RT (radiation-treated), ZBP1-KO1/KO2 (knockout clones).). (TIF) [file pgen.1012107.s007.tif]

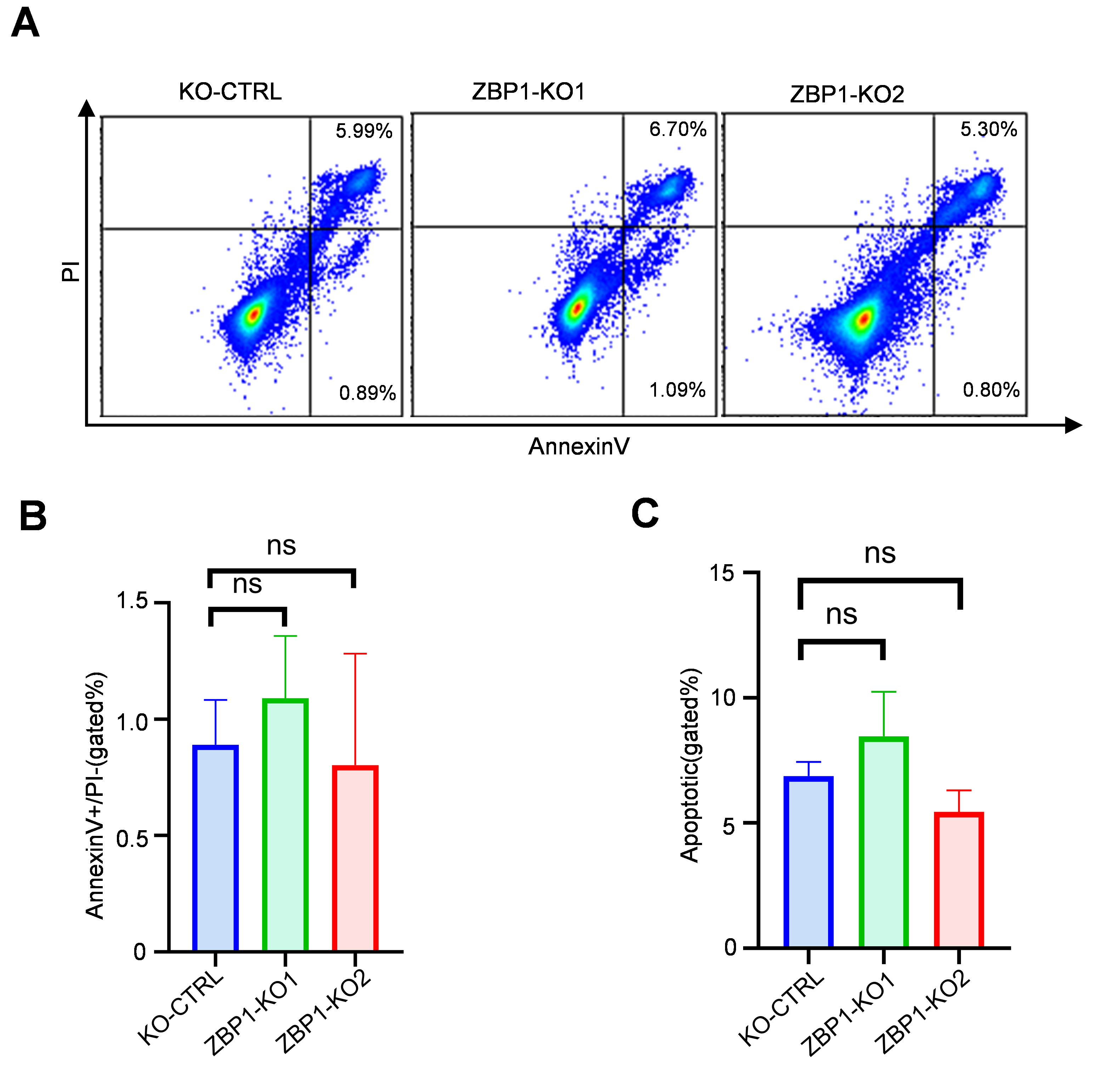

Supplement: S8 Fig — (A) Flow cytometry analysis of apoptosis in SCC-7 cells (KO-Ctrl vs two ZBP1-KO clones). (B) Quantification of early apoptotic cells (Annexin V + PI−) (C) Quantification of total apoptotic cells (Annexin V + PI+ plus Annexin V + PI−) (No significant differences [n.s.] by one-way ANOVA, n = 3 independent experiments; Group labels: CTRL (empty vector), RT (radiation-treated), ZBP1-KO1/KO2 (knockout clones).). (TIF) [file pgen.1012107.s008.tif]

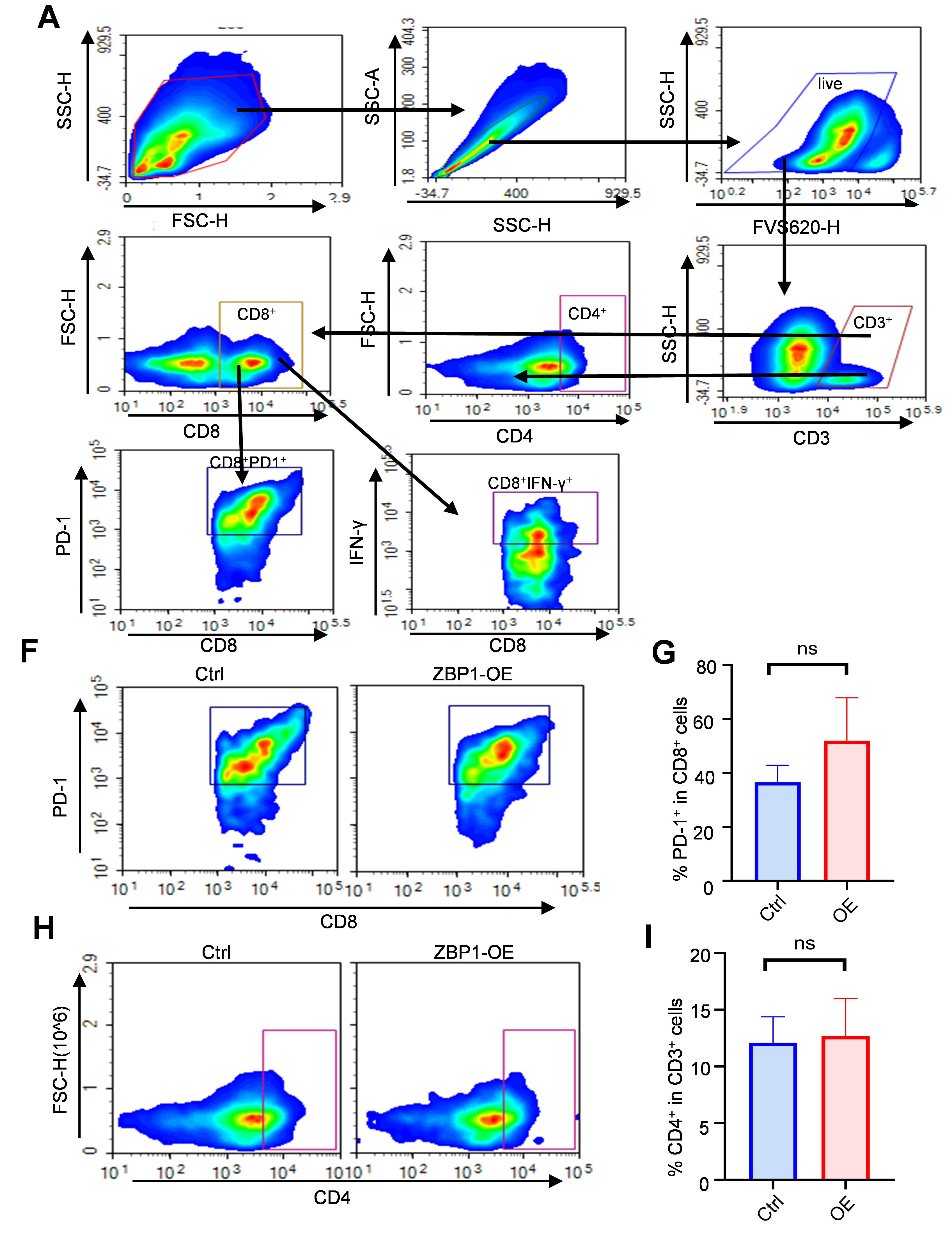

Supplement: S9 Fig — (A) Flow cytometry gating strategy for T cell detection in subcutaneous tumors. (B-C) CD8 + PD-1 + T cell infiltration (No significant differences [n.s.] by Student’s t-test). (D-E) CD4 + T cell infiltration (No significant differences [n.s.] by Student’s t-test). Group labels: Ctrl (control), OE(ZBP1-Overexpression). (TIF) [file pgen.1012107.s009.tif]

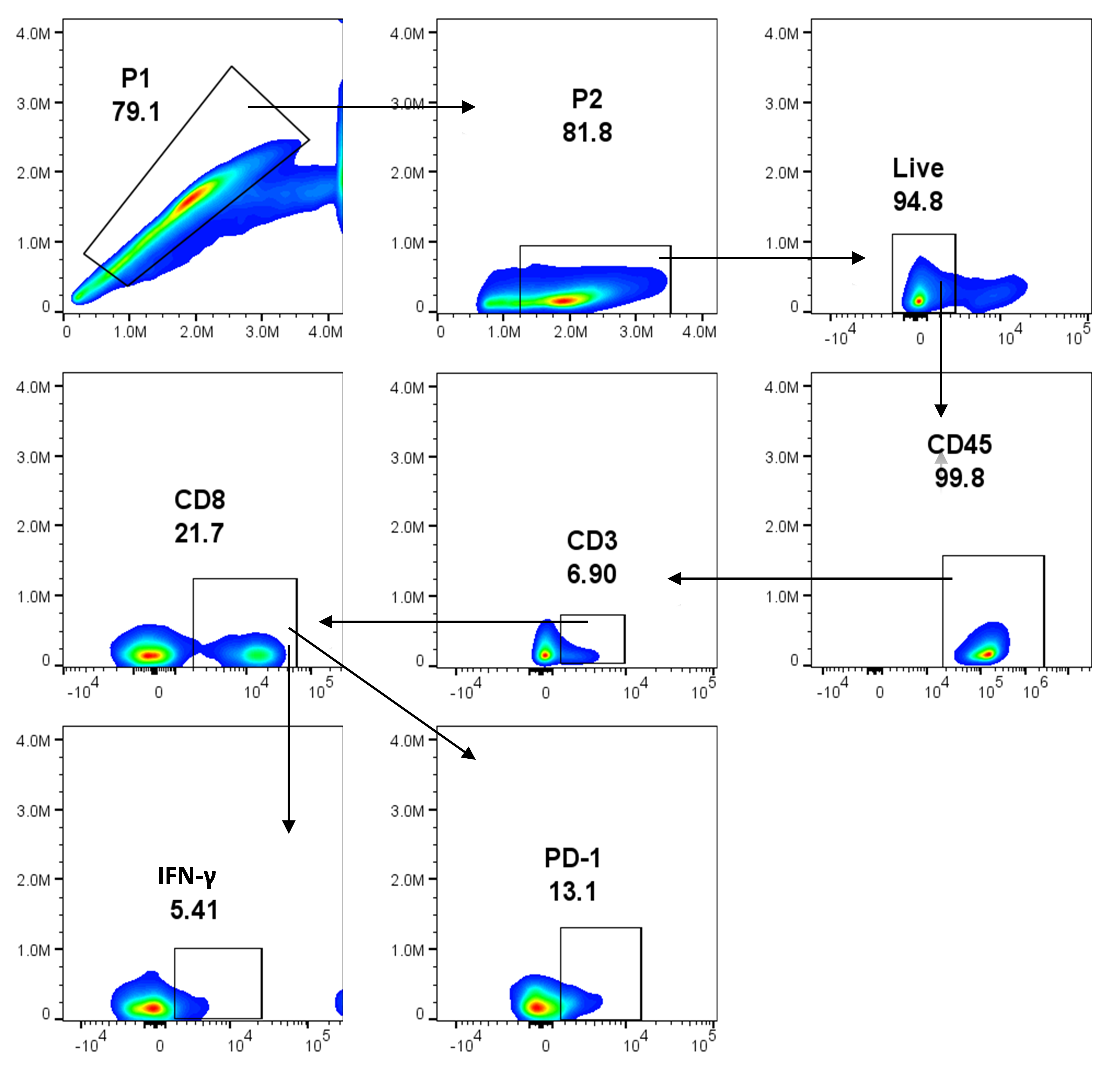

Supplement: S10 Fig — Representative gating strategy for identifying primary CD8 + T cells in the co-culture system. (TIF) [file pgen.1012107.s010.tif]

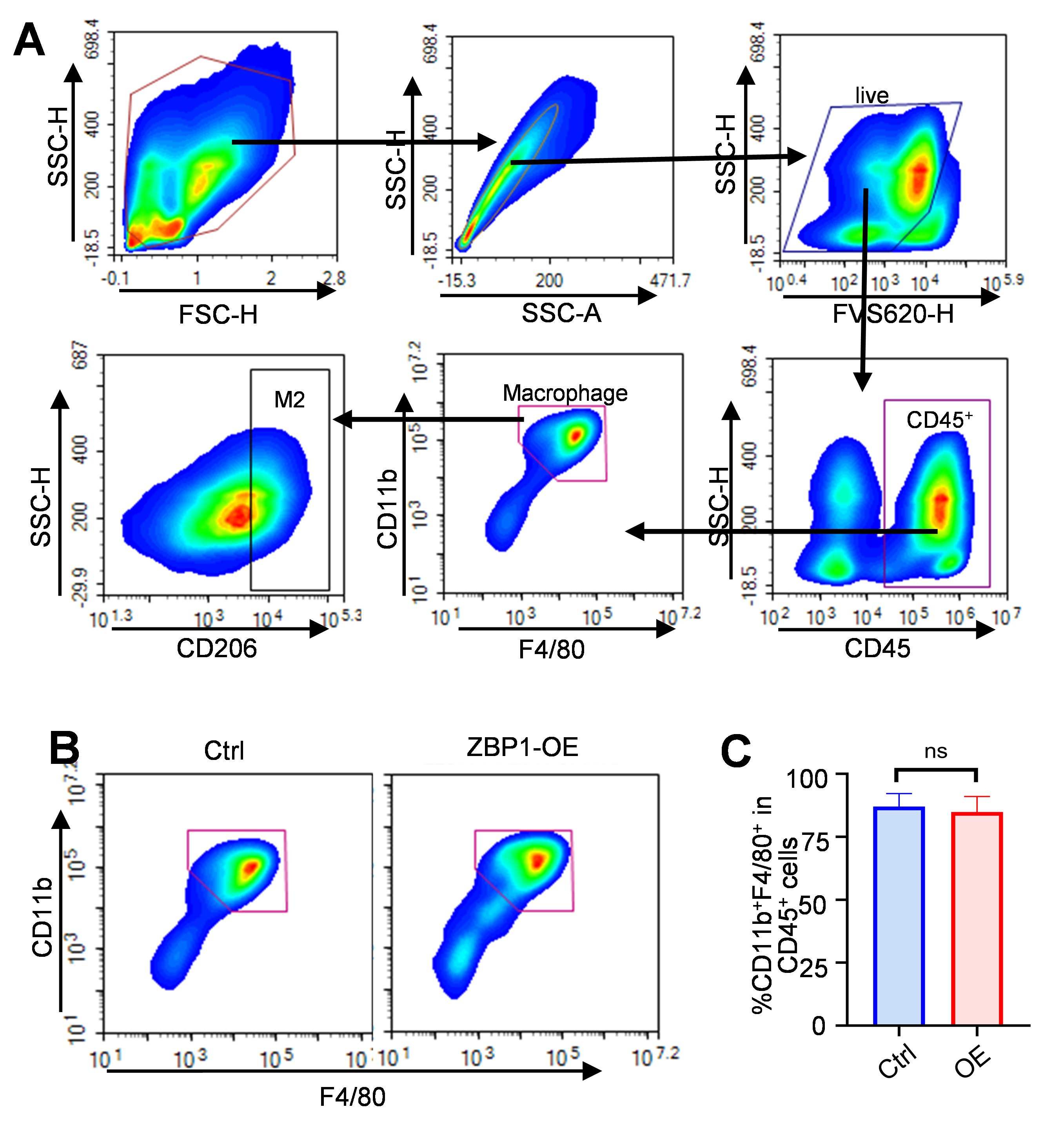

Supplement: S11 Fig — (A) Gating strategy for TAMs identification in subcutaneous tumor models. (B-C) CD11b+F480 +cell proportions. (No significant differences [n.s.] by Student’s t-test, n = 3 independent experiments; Group labels: Ctrl (control), OE(ZBP1-Overexpression)). (TIF) [file pgen.1012107.s011.tif]

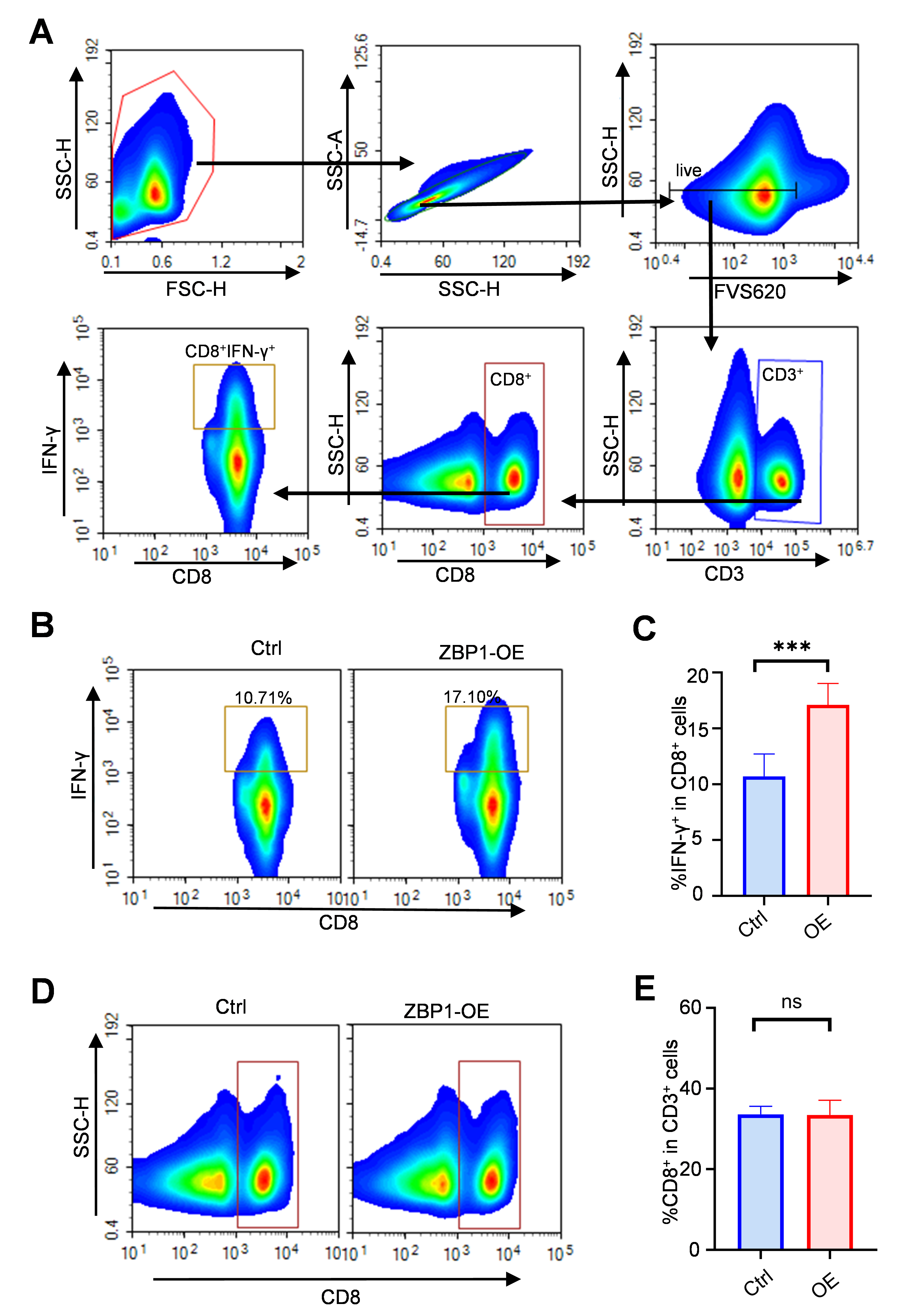

Supplement: S12 Fig — (A) Gating strategy for splenic T cell identification in subcutaneous tumor models. (B-C) IFN-γ + CD8 + T cell proportions within total CD8 + T cells (***p < 0.001 vs Ctrl, Student’s t-test). (D-E) CD8 + T cell frequency among CD3 + T cells (No significant differences [n.s.] by Student’s t-test. Group labels: Ctrl (control). (TIF) [file pgen.1012107.s012.tif]

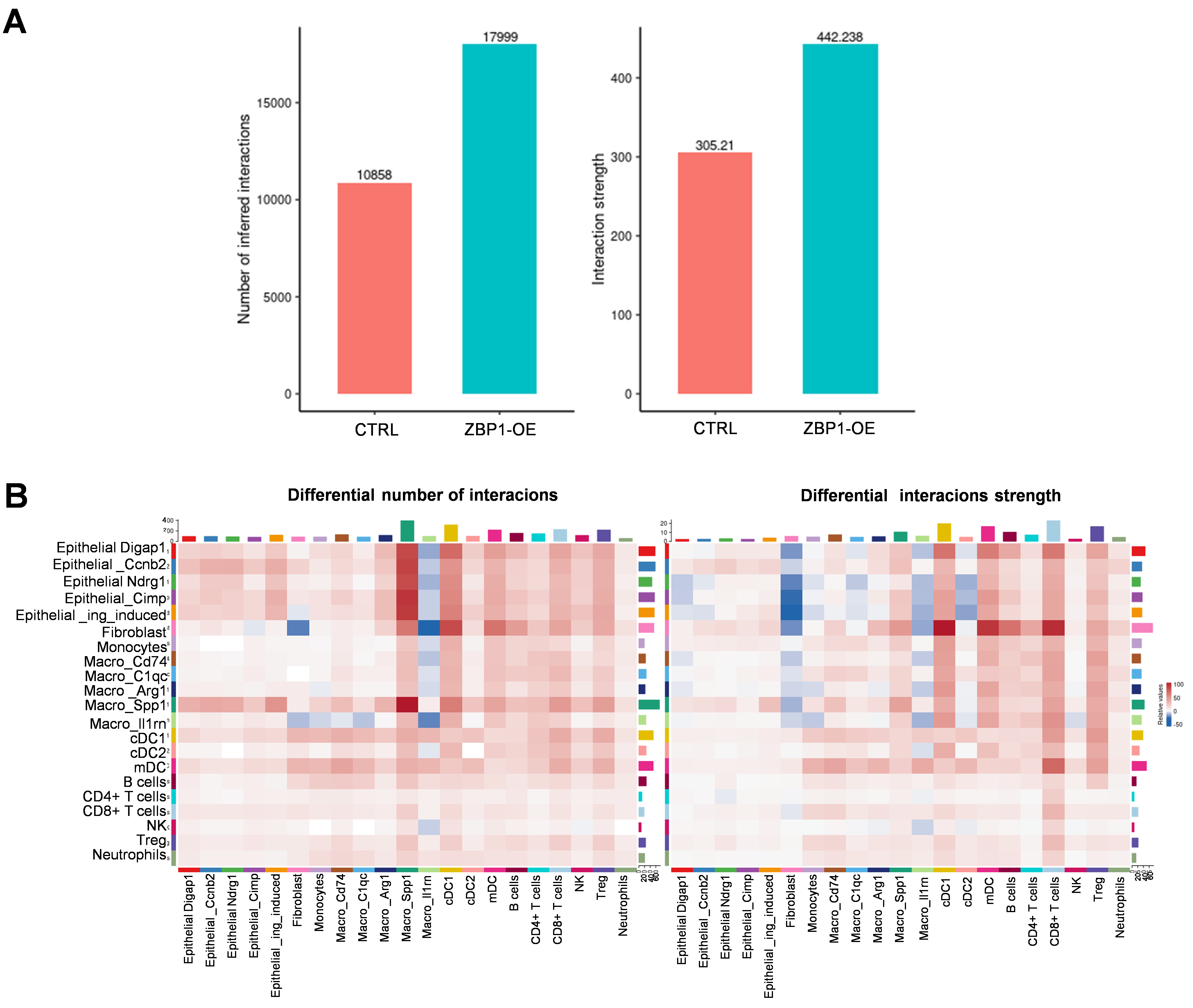

Supplement: S13 Fig — (A) Left panel: Quantitative comparison of ligand-receptor interaction numbers (bar plot, left) and interaction strength (bar plot, right) between control (CTRL) and ZBP1-overexpressing (ZBP1-OE) tumors. (B) Top panel: Heatmap of cell-cell interaction counts (left) and strength (right) across different subsets. (TIF) [file pgen.1012107.s013.tif]

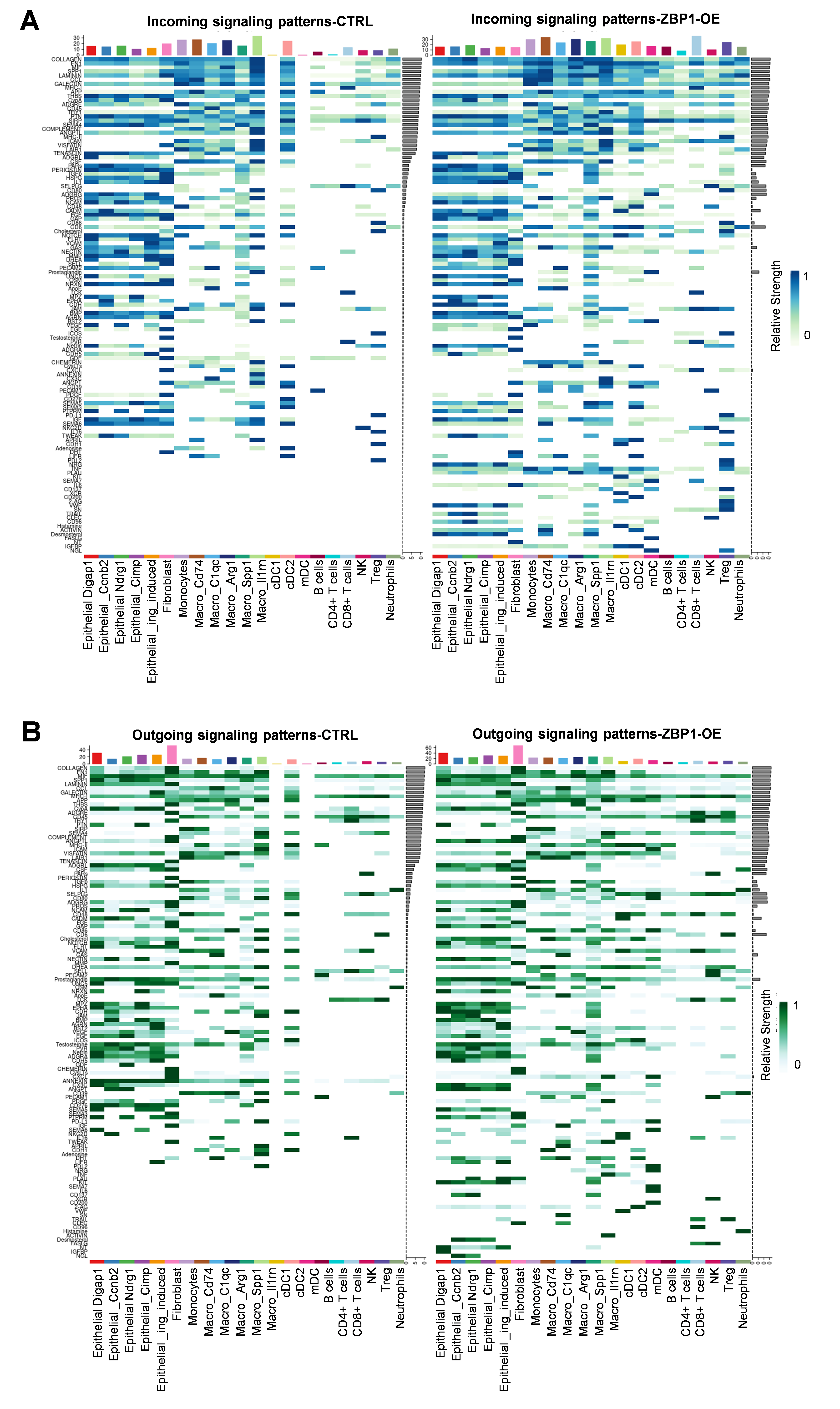

Supplement: S14 Fig — (A) Incoming signaling patterns in ZBP1-OE vs. CTRL different subsets. Heatmap rows: incoming pathways; columns: cell clusters. Color scale: normalized enrichment scores (NES, Z-score). (B) Outgoing signaling patterns from ZBP1-OE different subsets. Heatmap rows: outgoing pathways; columns: target cell types. Color scale: Z-scored interaction strength. (TIF) [file pgen.1012107.s014.tif]
