# Supplementary material for: ZBP1 Drives CD8+ T cell-mediated anti-tumor immunity in head and neck squamous cell carcinoma
Source: PLoS Genet. 2026 May 26;22(5):e1012107. doi: 10.1371/journal.pgen.1012107 (PMC13249162; doi:10.1371/journal.pgen.1012107)
Supplement: S1 Table — List of key reagents, including cell culture media, antibodies, assay kits, and critical chemicals, with corresponding manufacturers and countries of origin. (DOCX) [file pgen.1012107.s015.docx]

**Supplementary Table S1.**

| Name | Biotech Inc |
| --- | --- |
| DMEM | Gibco，America |
| Serun | Zeta life，America |
| Penicillin-Streptomycin Solution | Gibco，America |
| DMSO | MP Biomedicals，America |
| Trizol cell lysate | Invitrogen，America |
| RIPA cell lysate | Beyotime，China |
| chloroform | KESHI，Chengdu Kelong Chemical Co., Ltd |
| Isopropanol | KESHI，Chengdu Kelong Chemical Co., Ltd |
| Methanol | KESHI，Chengdu Kelong Chemical Co., Ltd |
| 75% ethanol | KESHI，Chengdu Kelong Chemical Co., Ltd |
| Xylene | KESHI，Chengdu Kelong Chemical Co., Ltd |
| Absolute ethanol | KESHI，Chengdu Kelong Chemical Co., Ltd |
| Protease inhibitors | Yazyme, China |
| Nuclease inhibitors | Sino China |
| Skim milk powder | Yili, China |
| 4% paraformaldehyde | KESHI，Chengdu Kelong Chemical Co., Ltd |
| Enhanced ChemiLuminescence | Merck KGaA, Germany |
| BCA kits | ThermoFisher Scientific，America |
| DNA extraction kits | TIANGEN，China |
| T7EI kits | Beyotime，China |
| Plasmid mini-extraction kit | TIANGEN，Beyotime，China |
| Glue recovery kit | Omega Biotek，美国 |
| ZBP1 antibody | Proteintech， China |
| CK14 antibody | Proteintech，China |
| CD8 antibody | Proteintech，China |
| CD4 antibody | Labvision，America |
| CD68 antibody | Labvision，America |
| CD11b antibody | Labvision，America |
| GAPDH antibody | ABclonal，China |
| Horseradish enzyme labeling goat anti-mouse IgG (H^+^L) | Ch Beijing Zhongshan Golden Bridge Biotechnology Co. Ltd. |
| Horseradish enzyme labeling goat anti-rabbit IgG (H^+^L) | Ch Beijing Zhongshan Golden Bridge Biotechnology Co. Ltd. |
| Goat anti-mouse fluorescent secondary antibody 488 | Invitrogen，America |
| Goat anti-rabbit fluorescent secondary antibody 568 | Invitrogen，America |
| DAPI | Beyotime，China |
| Neutral resin | Solarbio，China |
| Flow cytometry antibodies CD45-APC | BioLegend，America |
| Flow cytometry antibodies F4/80-FITC | BioLegend，America |
| Flow cytometry antibodies CD206-PE/Cy7 | BioLegend，America |
| Flow cytometry antibodies CD11b-FITC | BioLegend，America |
| Flow cytometry antibodies MHC-II-PE | BioLegend，America |
| Flow cytometry antibodies CD80-PE-Cy7 | BioLegend，America |
| Flow cytometry antibodies CD86-APC-Cy7 | BioLegend，America |
| Flow cytometry antibodies CD3-APC | BioLegend，America |
| Flow cytometry antibodies CD4-FITC | BioLegend，America |
| Flow cytometry antibodies CD8-APC-Cy7 | BioLegend，America |
| Flow cytometry antibodies PD-1-PE/Cy7 | BioLegend，America |
| Flow cytometry antibodies INF-γ-PE | BioLegend，America |
| FC-block | Bio x cell，America |
| FVS 620 | Bd biosciences，America |
| Apoptosis assay kits | Yeasen Biotechnology，China |
| CCK8 cell staining reagent | Hanheng Biotech,，China |
| Crystal violet staining reagent | Beyotime，China |
| Immunohistochemistry staining kits | Ch Beijing Zhongshan Golden Bridge Biotechnology Co. Ltd. |
| Multicolor immunofluorescence kits | Panovue Biotechnology (Beijing) Co., Ltd |
| Hematoxylin | Beyotime，China |
| Antifluorescence quenching mountant | Panovue Biotechnology (Beijing) Co., Ltd |
| qRT-PCR kit | Takara，Japan |
