# Supplementary material for: ZBP1 Drives CD8+ T cell-mediated anti-tumor immunity in head and neck squamous cell carcinoma
Source: PLoS Genet. 2026 May 26;22(5):e1012107. doi: 10.1371/journal.pgen.1012107 (PMC13249162; doi:10.1371/journal.pgen.1012107)
Supplement: S1 Text — (PDF) [file pgen.1012107.s016.pdf]

Detection Result

(1) Basic Inspection Information

Number	Polyallelic base Follow	Control cell line	Human contamination	Compared with control cells Distribution EV value	Match Description
20230228-01	Not have	SCC-7	Not have	0.95	Basic match

Sample genotype test results

- Polyallelic refers to the phenomenon of three or more alleles.
- The cytometric analysis results were satisfactory for all cell subtypes in this study.

(II) Sample Descriptions

- 20230228-01: The cell strain was identified as **mouse cell line**, which was **basically matched with** the control mouse cell line **SCC-7**.**No polialleles were detected** in this cell line.
- **Note:**The test cell lines were compared with STR data of cell lines included in ATCC, DSMZ, JCRB, and RIKEN databases. Cell lines not included in these databases will not be matched. Among the following loci,D4S2408 is a human-derived locus used to detect human contamination in the cells.

(Three)Sample typing results **Genotyping results of STR loci and Amelogenin locus in cell 20230228-01**

Loci	Sent cell STR information				Cell bank cell STR information		
	Sent cell name: SCC-7				Cell bank cell name: SCC-7		
	Allele1	Allele2	Allele3	Allele4	Allele1	Allele2	Allele3
4-2	230.28 [18.3]	234.29 [19.3]			18.3	19.3	
5-5	336.11 [14]				14		
6-4	300.49 [18]				18		
6-7	334.96 [12]				12		
9-2	221.77 [15]						
12-1	226.5 [16]				16		
15-3	209.45 [24.3]	213.55 [25.3]			25.3		
18-3	152.91 [16]				16		
X-1	408.88 [27]				27		
D4S2408							
