# Supplementary material for: ZBP1 Drives CD8+ T cell-mediated anti-tumor immunity in head and neck squamous cell carcinoma
Source: PLoS Genet. 2026 May 26;22(5):e1012107. doi: 10.1371/journal.pgen.1012107 (PMC13249162; doi:10.1371/journal.pgen.1012107)
Supplement: S2 Text — (PDF) [file pgen.1012107.s017.pdf]

STR Cell Line Identification Report

Commissioned platform: Genomics Platform of Xinchuan Public Experimental Technology Center

Sample provider: Julie Li	Sample submission date: 2023.05.24	Project Leader: Dai Lei
Appraiser: Guo Zhouhong/Liu Ziyi	Sample type: Cell	Cell name: MOC1

Test method：

DNA extraction: Magpure Tissue & Blood DNA LQ Kit (Magen)

Site amplification reagent: Takara R007

Sequencer: Thermo 3730XL

size standard： genescan liz 500

Classification result:

Loci	Allele1	Allele2	Allele3
15-3	22.3		
9-2	18		
6-4	18		
6-7	15		
18-3	16		
4-2	20.3		
5-5	15		
12-1	19	20	
x-1	27		

Sequencing peak profile:

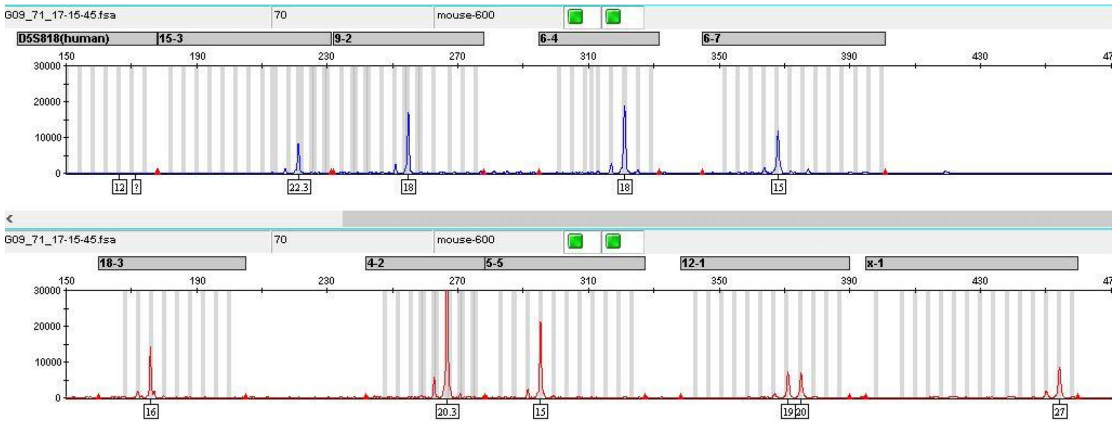

Database comparison results

Number	Matched cell lines	Match degree	Data base	Is it contaminated?	Cell Line Evaluation
20230601-71			Expasy	Deny	Not in line with expectations

Note: The MOC1 cell line lacks STR data in domestic and international databases, and the submitted cell line could not be matched with any other cell line in the databases.

If you have any questions, please contact 584537011@qq.com or WeChat: 584537011 Tel: 13281296192

For papers that utilize central instruments or technical guidance services to complete testing or technical outputs, please indicate in the acknowledgments section: "Acknowledgments to XX Instrument Group or Professor XX (XX, Core Facilities of West China Hospital) at the Public Laboratory Technology Center of West China Hospital."

STR Cell Line Identification Report

Commissioned platform: Genomics Platform of Xinchuan Public Experimental Technology Center

Sample provider: Julie Li	Sample submission date: 2023.05.24	Project Leader: Dai Lei
Appraiser: Guo Zhouhong/Liu Ziyi	Sample type: Cell	Cell name: MOC2

Test method：

DNA extraction: Magpure Tissue & Blood DNA LQ Kit (Magen)

Site amplification reagent: Takara R007

Sequencer: Thermo 3730XL

size standard： genescan liz 500

Classification result:

Loci	Allele1	Allele2	Allele3
15-3	22.3		
9-2	18		
6-4	18		
6-7	15		
18-3	16		
4-2	20.3		
5-5	15		
12-1	19	20	
x-1	27		

Sequencing peak profile:

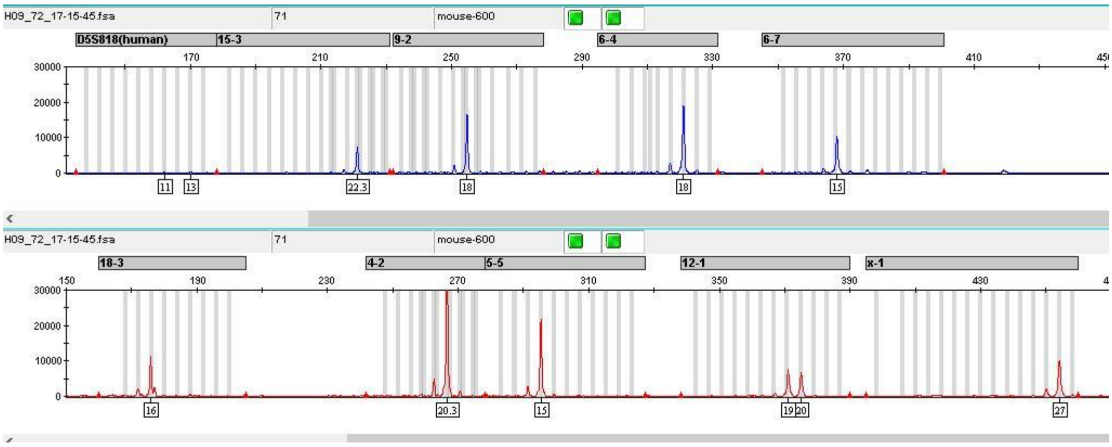

Database comparison results

Number	Matched cell lines	Match degree	Data base	Is it contaminated?	Cell Line Evaluation
20230601-71			Expasy	deny	

Note: The MOC2 cell line lacks STR data in domestic and international databases, and the submitted cell line could not be matched with any other cell line in the databases.

If you have any questions, please contact 584537011@qq.com or WeChat: 584537011 Tel: 13281296192

For papers that utilize central instruments or technical guidance services to complete testing or technical outputs, please indicate in the acknowledgments section: "Acknowledgments to XX Instrument Group or Professor XX (XX, Core Facilities of West China Hospital) at the Public Laboratory Technology Center of West China Hospital."
