# Supplementary material for: ZBP1 Drives CD8+ T cell-mediated anti-tumor immunity in head and neck squamous cell carcinoma
Source: PLoS Genet. 2026 May 26;22(5):e1012107. doi: 10.1371/journal.pgen.1012107 (PMC13249162; doi:10.1371/journal.pgen.1012107)
Supplement: S3 Text — (PDF) [file pgen.1012107.s018.pdf]

STR Cell Line Identification Report

Commissioned platform: Genomics Platform of Xinchuan Public Experimental Technology Center

Sample provider: He Ling	Sample submission date: 2023.05.29	Project Leader: Dai Lei
Appraiser: Guo Zhouhong/Liu Ziyi	Sample type: Cell	Cell name: FaDu

Test method：

DNA extraction: Magpure Tissue & Blood DNA LQ Kit (Magen)

Site amplification reagent: Takara R007

Sequencer: Thermo 3730XL

size standard：genescan liz 500

Classification result:

Loci	Allele1	Allele2	Allele3
D5S818	12	13	
TH01	8		
D13S317	8	9	
D16S539	11		
vWA	15	17	
TPOX	11		
D7S820	11	12	
CSF1PO	12		
Amel			

Sequencing peak profile:

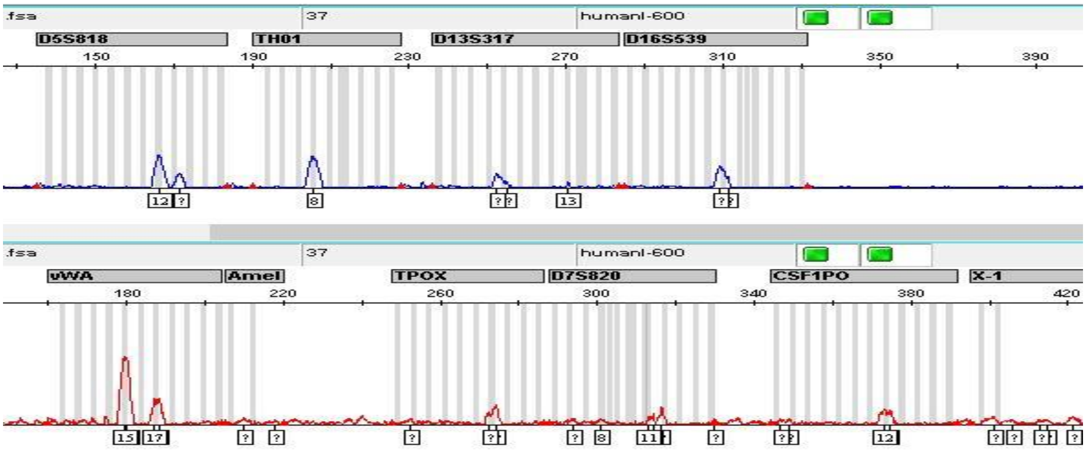

Database comparison results

Number	Matched cell lines	Match degree	Data base	Is it contaminated?	Cell Line Evaluation
20230601-37	FaDu	95.65%	Expasy	Deny	Be in line with forecast

If you have any questions, please contact 584537011@qq.com or WeChat: 584537011 Tel: 13281296192

For papers that utilize central instruments or technical guidance services to complete testing or technical outputs, please indicate in the acknowledgments section: "Acknowledgments to XX Instrument Group or Professor XX (XX, Core Facilities of West China Hospital) at the Public Laboratory Technology Center of West China Hospital."
